# Supplementary material for: Impact of youth lay health workers on HIV service delivery in South Africa: A pragmatic cluster randomized trial of the Youth Health Africa program
Source: PLoS One. 2023 Nov 30;18(11):e0294719. doi: 10.1371/journal.pone.0294719 (PMC10688901; doi:10.1371/journal.pone.0294719)
Supplement: S1 Appendix — (PDF) [file pone.0294719.s001.pdf]

Manuscript: Impact of youth lay health workers on HIV service delivery in South Africa: a pragmatic cluster randomized trial of the Youth Health Africa program

SUPPLEMENT 1: CONSORT CHECKLIST

CONSORT Reporting Guidelines

		Reporting Item	Page Number
**Title and Abstract**			
Title	#1a	Identification as a randomized trial in the title.	1
Abstract	#1b	Structured summary of trial design, methods, results, and conclusions	2
**Introduction**			
Background and objectives	#2a	Scientific background and explanation of rationale	3
Background and objectives	#2b	Specific objectives or hypothesis	3
**Methods**			
Trial design	#3a	Description of trial design (such as parallel, factorial) including allocation ratio.	3-4 (Section 2.1, 2.3)
Trial design	#3b	Important changes to methods after trial commencement (such as eligibility criteria), with reasons	N/A
Participants	#4a	Eligibility criteria for participants	5 (Section 2.4)
Participants	#4b	Settings and locations where the data were collected	4 (Section 2.2)
Interventions	#5	The experimental and control interventions for each group with sufficient details to allow replication, including how and when they were actually administered	4 (Section 2.3)
Outcomes	#6a	Completely defined prespecified primary and secondary outcome measures, including how and when they were assessed	5-6 (Section 2.5)
Outcomes	#6b	Any changes to trial outcomes after the trial commenced, with reasons	N/A
Sample size	#7a	How sample size was determined.	5 (Section 2.4), 9 (Section 2.9)
Sample size	#7b	When applicable, explanation of any interim analyses and stopping guidelines	N/A
Randomization - Sequence generation	#8a	Method used to generate the random allocation sequence.	5 (2.4)
Randomization - Sequence generation	#8b	Type of randomization; details of any restriction (such as blocking and block size)	5 (2.4)
Randomization - Allocation concealment mechanism	#9	Mechanism used to implement the random allocation sequence (such as sequentially numbered containers), describing any steps taken to conceal the sequence until interventions were assigned	5 (2.4)
Randomization - Implementation	#10	Who generated the allocation sequence, who enrolled participants, and who assigned participants to interventions	5 (2.4)
Blinding	#11a	If done, who was blinded after assignment to interventions and how.	N/A (Section 2.4 – No blinding)

Blinding	#11b	If relevant, description of the similarity of interventions	N/A
Statistical methods	#12a	Statistical methods used to compare groups for primary and secondary outcomes	6-9 (Section 2.7. 2.8)
Statistical methods	#12b	Methods for additional analyses, such as subgroup analyses and adjusted analyses	6-9 (Section 2.7. 2.8)
**Results**			
Participant flow diagram (strongly recommended)	#13a	For each group, the numbers of participants who were randomly assigned, received intended treatment, and were analysed for the primary outcome	10 (Sections 3.1), Figure 1
Participant flow	#13b	For each group, losses and exclusions after randomization, together with reason	10 (Section 3.1)
Recruitment	#14a	Dates defining the periods of recruitment and follow-up	10 (Section 3.1)
Recruitment	#14b	Why the trial ended or was stopped	4 (Section 2.1)
Baseline data	#15	A table showing baseline demographic and clinical characteristics for each group	11 (Table 2)
Numbers analysed	#16	For each group, number of participants (denominator) included in each analysis and whether the analysis was by original assigned groups	10 (Section 3.1 and Figure 1)
Outcomes and estimation	#17a	For each primary and secondary outcome, results for each group, and the estimated effect size and its precision (such as 95% confidence interval)	11-13 (Sections 3.3, 3,4)
Outcomes and estimation	#17b	For binary outcomes, presentation of both absolute and relative effect sizes is recommended	N/A
Ancillary analyses	#18	Results of any other analyses performed, including subgroup analyses and adjusted analyses, distinguishing pre-specified from exploratory	13-14 (Section 3.5)
Harms	#19	All important harms or unintended effects in each group (For specific guidance see CONSORT for harms)	N/A
**Discussion**			
Limitations	#20	Trial limitations, addressing sources of potential bias, imprecision, and, if relevant, multiplicity of analyses	17 (Section 4.1)
Generalisability	#21	Generalisability (external validity, applicability) of the trial findings	18 (Section 4.2)
Interpretation	#22	Interpretation consistent with results, balancing benefits and harms, and considering other relevant evidence	13-18
Registration	#23	Registration number and name of trial registry	9 (Section 2.10)
**Other information**			
Protocol	#24	Where the full trial protocol can be accessed, if available	1 (Title page)
Funding	#25	Sources of funding and other support (such as supply of drugs), role of funders	1 (Title page)

None The CONSORT checklist is distributed under the terms of the Creative Commons Attribution License CC-BY. This checklist can be completed online using https://www.goodreports.org/, a tool made by the EQUATOR Network in collaboration with Penelope.ai
