# Supplementary material for: Impact of youth lay health workers on HIV service delivery in South Africa: A pragmatic cluster randomized trial of the Youth Health Africa program
Source: PLoS One. 2023 Nov 30;18(11):e0294719. doi: 10.1371/journal.pone.0294719 (PMC10688901; doi:10.1371/journal.pone.0294719)
Supplement: S2 Appendix — (PDF) [file pone.0294719.s002.pdf]

SUPPLEMENT 2: TRIAL MONITORING DATA

Figure S2.1. Intern placements over time (Intention to Treat)

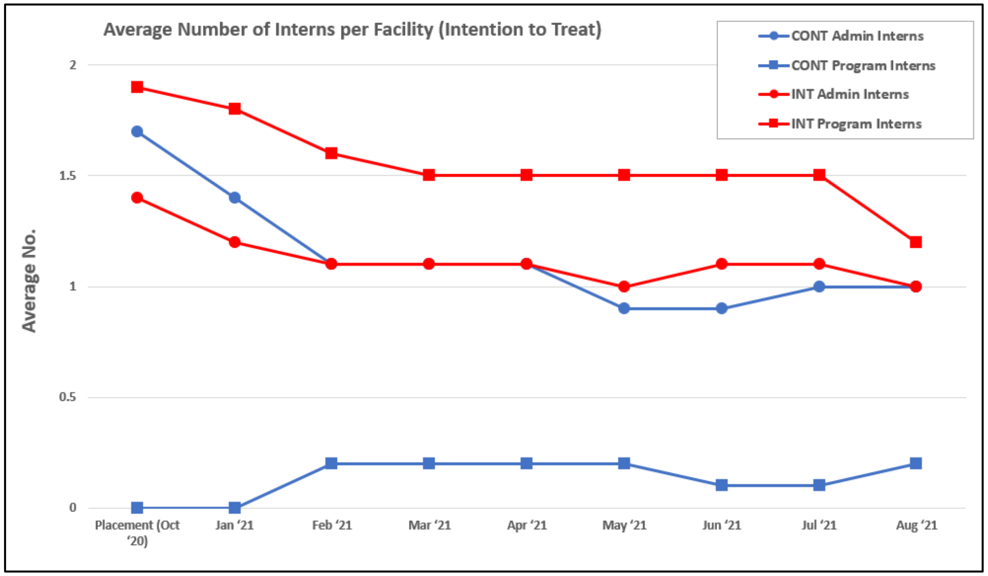

Figure S2.2. Intern placements over time (As Treated)

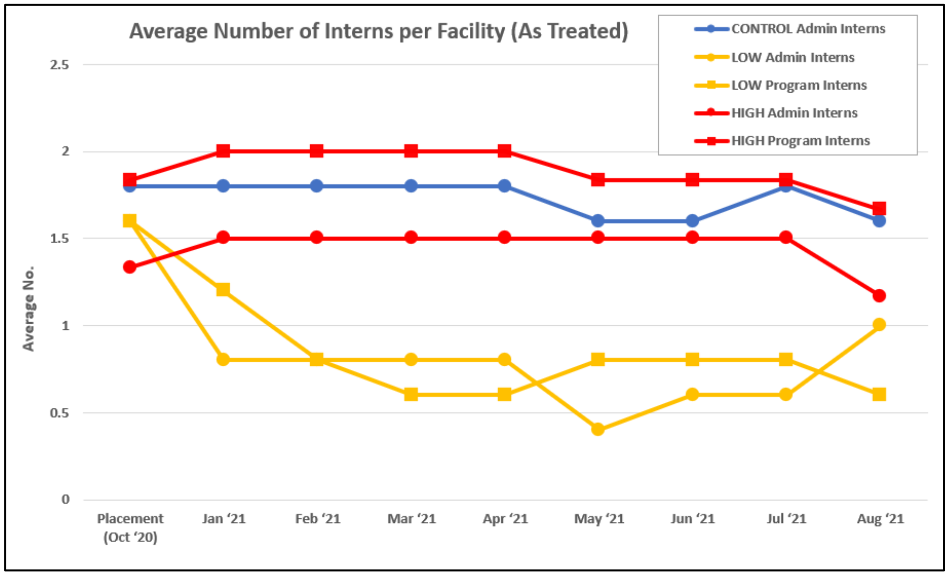

Table S2.1. Average number of interns in place at clinics participating in trial (intention to treat analysis)

		Placement (Oct '20)	Jan '21	Feb '21	Mar '21	Apr '21	May '21	Jun '21	Jul '21	Aug '21
CONTROL (N=10)	Admin Interns	1.7	1.4	1.1	1.1	1.1	0.9	0.9	1	1
	Program Interns	0	0	0.2	0.2	0.2	0.2	0.1	0.1	0.2
INTERVENTION (N=10)	Admin Interns	1.4	1.2	1.1	1.1	1.1	1	1.1	1.1	1
	Program Interns	1.9	1.8	1.6	1.5	1.5	1.5	1.5	1.5	1.2

Table S2.2. Average number of interns in place at clinics participating in trial (as treated analysis)

		Placement (Oct '20)	Jan '21	Feb '21	Mar '21	Apr '21	May '21	Jun '21	Jul '21	Aug '21
CONTROL (N=5)	Admin Interns	1.8	1.8	1.8	1.8	1.8	1.6	1.6	1.8	1.6
LOW INTERVENTION (N=5)	Admin Interns	1.6	0.8	0.8	0.8	0.8	0.4	0.6	0.6	1
	Program Interns	1.6	1.2	0.8	0.6	0.6	0.8	0.8	0.8	0.6
HIGH INTERVENTION (N=6)	Admin Interns	1.3	1.5	1.5	1.5	1.5	1.5	1.5	1.5	1.2
	Program Interns	1.8	2.0	2.0	2.0	2.0	1.8	1.8	1.8	1.7

Table S2.3: Number and proportion of months that facilities had interns during study period (Jan–Aug 2021) and the corresponding "as treated" assignment per facility.

		Number Months				% of Months				
Facility	ITT Group	Only A	A&P at same time	Only P	No interns	Only A	A&P at same time	Only P	No interns	As Treated Analysis
Facility 1	Con	8	0	0	0	100%	0%	0%	0%	Control
Facility 2	Con	8	0	0	0	100%	0%	0%	0%	Control
Facility 3	Con	8	0	0	0	100%	0%	0%	0%	Control
Facility 4	Con	6	0	0	2	75%	0%	0%	25%	Control
Facility 5	Con	8	0	0	0	100%	0%	0%	0%	Control
Facility 6	Con	0	0	0	8	0%	0%	0%	100%	Exclude
Facility 7	Con	1	0	0	7	13%	0%	0%	88%	Exclude
Facility 8	Con	1	0	7	0	13%	0%	88%	0%	Exclude
Facility 9	Con	1	0	4	3	13%	0%	50%	38%	Exclude
Facility 10	Con	7	1	0	0	88%	13%	0%	0%	Low
Facility 11	Int	0	8	0	0	0%	100%	0%	0%	High
Facility 12	Int	0	8	0	0	0%	100%	0%	0%	High
Facility 13	Int	0	7	1	0	0%	88%	13%	0%	High
Facility 14	Int	0	2	6	0	0%	25%	75%	0%	Low
Facility 15	Int	0	1	7	0	0%	13%	88%	0%	Low
Facility 16	Int	0	8	0	0	0%	100%	0%	0%	High
Facility 17	Int	0	8	0	0	0%	100%	0%	0%	High
Facility 18	Int	0	8	0	0	0%	100%	0%	0%	High
Facility 19	Int	4	3	1	0	50%	38%	13%	0%	Low
Facility 20	Int	6	1	1	0	75%	13%	13%	0%	Low

Con = Control; Int = Intervention; A = Admin intern; P = Program intern

Table S2.4. Monthly number of interns at facility – Measures of Central Tendency

	Mean	Median	Mode	Range
**Intention to Treat**				
**Control**	1.2	1	2	0-2
**Intervention**	2.6	3	3	1-5
**As Treated**				
**Control**	1.7	2	2	0-2
**High Intervention**	3.4	3	3	1-5
**Low Intervention**	1.5	1	1	1-3

Table S2.6. Total number of interns per month, per facility – Control Group

Facility	Intern Role	Oct '20	Jan '21	Feb '21	Mar '21	Apr '21	May '21	Jun '21	Jul '21	Aug '21
**CONTROL CLINICS**										
Facility 1	Admin	2	2	2	2	2	2	2	2	2
	Program	0	0	0	0	0	0	0	0	0
Facility 2	Admin	2	2	2	2	2	2	2	2	1
	Program	0	0	0	0	0	0	0	0	0
Facility 3	Admin	2	2	2	2	2	2	2	2	2
	Program	0	0	0	0	0	0	0	0	0
Facility 4	Admin	1	1	1	1	1	0	0	1	1
	Program	0	0	0	0	0	0	0	0	0
Facility 5	Admin	2	2	2	2	2	2	2	2	2
	Program	0	0	0	0	0	0	0	0	0
Facility 6	Admin	2	0	0	0	0	0	0	0	0
	Program	0	0	0	0	0	0	0	0	0
Facility 7	Admin	2	1	0	0	0	0	0	0	0
	Program	0	0	0	0	0	0	0	0	0
Facility 8	Admin	1	2	0	0	0	0	0	0	0
	Program	0	0	1	1	1	1	1	1	1
Facility 9	Admin	1	1	0	0	0	0	0	0	0
	Program	0	0	1	1	1	1	0	0	0
Facility 10	Admin	2	1	2	2	2	1	1	1	2
	Program	0	0	0	0	0	0	0	0	1
**TOTALS**	**Admin**	**17**	**14**	**11**	**11**	**11**	**9**	**9**	**10**	**10**
	**Program**	**0**	**0**	**2**	**2**	**2**	**2**	**1**	**1**	**2**
**All Control Interns**		**17**	**14**	**13**	**13**	**13**	**11**	**10**	**11**	**12**

Table S2.7. Total number of interns per month, per facility – Intervention Group

Facility	Intern Role	Oct '20	Jan '21	Feb '21	Mar '21	Apr '21	May '21	Jun '21	Jul '21	Aug '21
**INTERVENTION CLINICS**										
Facility 11	Admin	1	1	1	1	1	1	1	1	1
	Program	2	2	2	2	2	2	2	2	2
Facility 12	Admin	2	2	1	1	1	1	1	1	2
	Program	2	2	2	2	2	1	1	1	2
Facility 13	Admin	1	2	2	2	2	2	2	2	0
	Program	1	2	3	3	3	3	3	3	1
Facility 14	Admin	1	1	0	0	0	0	0	0	1
	Program	2	1	1	1	1	1	1	1	1
Facility 15	Admin	2	1	0	0	0	0	0	0	0
	Program	2	2	2	2	2	2	2	2	1
Facility 16	Admin	1	1	2	2	2	2	2	2	2
	Program	2	2	1	1	1	1	1	1	1
Facility 17	Admin	2	2	1	1	1	1	1	1	1
	Program	2	2	2	2	2	2	2	2	2
Facility 18	Admin	1	1	2	2	2	2	2	2	1
	Program	2	2	2	2	2	2	2	2	2
Facility 19	Admin	2	0	1	1	1	1	1	1	1
	Program	2	2	1	0	0	0	1	1	0
Facility 20	Admin	1	1	1	1	1	0	1	1	1
	Program	2	1	0	0	0	1	0	0	0
**TOTALS**	**Admin**	**14**	**12**	**11**	**11**	**11**	**10**	**11**	**11**	**10**
	**Program**	**19**	**18**	**16**	**15**	**15**	**15**	**15**	**15**	**12**
**All Intervention Interns**		**33**	**30**	**27**	**26**	**26**	**25**	**26**	**26**	**22**
