# Supplementary material for: Impact of youth lay health workers on HIV service delivery in South Africa: A pragmatic cluster randomized trial of the Youth Health Africa program
Source: PLoS One. 2023 Nov 30;18(11):e0294719. doi: 10.1371/journal.pone.0294719 (PMC10688901; doi:10.1371/journal.pone.0294719)
Supplement: S3 Appendix — (PDF) [file pone.0294719.s003.pdf]

SUPPLEMENT 3: MODEL PARAMETERS FOR TIME SERIES ANALYSES (INTENTION TO TREAT)

Table S3. Model parameters for time series analysis using intention to treat groupings. Model for analysis was: $Y_{ijt} = \beta_0 + \beta_1 Time_{ijt} + \beta_2 Intervention_{ijt} + \beta_3 TimeAfterIntervention_{ijt} + \beta_4 Treat_{ijt} + B_5 Treat*Time_{ijt} + \beta_6 Treat*Intervention_{ijt} + \beta_7 Treat*TimeAfterIntervention_{ijt} + E_{ij}$

	Beta Estimate	Standard Error	95% CI: Lower	95% Upper	Significant (p<0.05)
**TESTING**					
**% Tested**					
β_0	17.9%	3.7%	10.7%	25.2%	*NA*
β_1Time	-0.2%	0.3%	-0.9%	0.5%	*NA*
β_3TimeAfterIntervention	0.8%	0.5%	-0.1%	1.7%	*NA*
β_2Intervention	2.2%	2.9%	-3.4%	7.8%	*NA*
β_4Treat	3.5%	5.2%	-7.5%	14.5%	*NA*
B_5Treat*Time	0.2%	0.5%	-0.7%	1.2%	*NA*
**β_6Treat*Intervention**	**8.4%**	**4.0%**	**0.5%**	**16.4%**	**YES (p=0.036)**
**β_7Treat*TimeAfterIntervention**	**-0.8%**	**0.6%**	**-2.0%**	**0.5%**	**NO**
**% HIV Pos**					
β_0	3.6%	0.9%	1.8%	5.4%	*NA*
β_1Time	0.0%	0.1%	-0.3%	0.2%	*NA*
β_3TimeAfterIntervention	-0.1%	0.2%	-0.4%	0.3%	*NA*
β_2Intervention	0.3%	1.2%	-2.1%	2.6%	*NA*
β_4Treat	-1.4%	1.3%	-4.1%	1.3%	*NA*
B_5Treat*Time	0.3%	0.2%	-0.1%	0.6%	*NA*
**β_6Treat*Intervention**	**-2.1%**	**1.7%**	**-5.4%**	**1.2%**	**NO**
**β_7Treat*TimeAfterIntervention**	**-0.3%**	**0.3%**	**-0.8%**	**0.2%**	**NO**
**INITIATED**					
**% Initiated within 14 days**					
β_0	51.1%	11.2%	29.2%	73.1%	*NA*
β_1Time	-1.2%	1.0%	-3.1%	0.7%	*NA*
β_3TimeAfterIntervention	-0.3%	1.4%	-3.1%	2.6%	*NA*
β_2Intervention	7.8%	8.9%	-9.7%	25.4%	*NA*
β_4Treat	12.9%	15.7%	-20.2%	45.9%	*NA*
B_5Treat*Time	1.0%	1.4%	-1.7%	3.7%	*NA*
**β_6Treat*Intervention**	**-10.8%**	**12.4%**	**-35.2%**	**13.5%**	**NO**
**β_7Treat*TimeAfterIntervention**	**0.7%**	**2.0%**	**-3.2%**	**4.6%**	**NO**

Continued on next page

Table S3 continued

	Beta Estimate	Standard Error	95% CI: Lower	95% Upper	Significant ($p<0.05$)
**RETAINED**					
**% Early Default (28 days)**					
β_0	10.2%	0.8%	8.6%	11.9%	*NA*
β_1Time	0.0%	0.1%	-0.2%	0.2%	*NA*
β_3TimeAfterIntervention	-0.2%	0.2%	-0.5%	0.1%	*NA*
β_2Intervention	1.2%	1.0%	-0.8%	3.2%	*NA*
β_4Treat	-0.6%	1.2%	-3.1%	1.9%	*NA*
B_5Treat*Time	-0.1%	0.2%	-0.4%	0.2%	*NA*
**β_6Treat*Intervention**	**0.5%**	**1.4%**	**-2.4%**	**3.3%**	**NO**
**β_7Treat*TimeAfterIntervention**	**0.1%**	**0.2%**	**-0.4%**	**0.5%**	**NO**
**% Late Default (90 days)**					
β_0	5.7%	0.8%	4.1%	7.4%	*NA*
β_1Time	0.0%	0.1%	-0.2%	0.1%	*NA*
β_3TimeAfterIntervention	-0.2%	0.2%	-0.5%	0.1%	*NA*
β_2Intervention	0.2%	0.8%	-1.3%	1.7%	*NA*
β_4Treat	-0.5%	1.2%	-3.0%	2.0%	*NA*
B_5Treat*Time	0.0%	0.1%	-0.2%	0.3%	*NA*
**β_6Treat*Intervention**	**-0.4%**	**1.1%**	**-2.5%**	**1.8%**	**NO**
**β_7Treat*TimeAfterIntervention**	**0.0%**	**0.2%**	**-0.4%**	**0.5%**	**NO**
**% ULTFU**					
β_0	14.3%	0.8%	12.8%	15.7%	*NA*
β_1Time	-0.7%	0.1%	-0.8%	-0.5%	*NA*
β_3TimeAfterIntervention	0.4%	0.1%	0.2%	0.7%	*NA*
β_2Intervention	-0.6%	0.3%	-1.2%	0.1%	*NA*
β_4Treat	-1.8%	1.1%	-4.0%	0.4%	*NA*
B_5Treat*Time	0.2%	0.1%	0.0%	0.4%	*NA*
**β_6Treat*Intervention**	**-0.4%**	**0.5%**	**-1.3%**	**0.5%**	**NO**
**β_7Treat*TimeAfterIntervention**	**-0.2%**	**0.2%**	**-0.6%**	**0.1%**	**NO**
