# Supplementary material for: Impact of youth lay health workers on HIV service delivery in South Africa: A pragmatic cluster randomized trial of the Youth Health Africa program
Source: PLoS One. 2023 Nov 30;18(11):e0294719. doi: 10.1371/journal.pone.0294719 (PMC10688901; doi:10.1371/journal.pone.0294719)

SUPPLEMENT 4. SENSITIVITY ANALYSIS – AS-TREATED RESULTS (HIGH INTERVENTION VS CONTROL FACILITIES)

Table S4.1. Comparison of change in HIV service indicators between control and high intervention facilities after implementation of Youth Health Africa (*Difference-in-Difference analysis*). The baseline period baseline period was January-August 2020. The study period was January-August 2021.

	CONTROL (n=5)<br>% (95% CI)			HIGH INTERVENTION (n=6)<br>% (95% CI)			Difference-in-Difference<br>% (95% CI)	P-value
	Baseline	Study	Difference	Baseline	Study	Difference		
**% Tested for HIV***	16.5%<br>(10.0–23.0%)	21.9%<br>(6.1–37.7%)	5.4%<br>(-3.9–14.6%)	22.9%<br>(7.5–38.3%)	34.5%<br>(-2.6–71.7%)	11.6%<br>(-10.2–33.4%)	6.2%<br>(-6.3–18.7%)	0.31
**% Positive for HIV**	3.0%<br>(2.1–3.8%)	2.2%<br>(0.2% - 4.1%)	-0.8%<br>(-1.9–0.4%)	2.3%<br>(0.4–4.2%)	1.7%<br>(-2.9–6.4%)	-0.6%<br>(-3.3–2.1%)	0.2%<br>(-1.4%, 1.8%)	0.81
**% Initiated on Txt within 14 days**	47.6%<br>(15.3–79.9%)	46.0%<br>(-31.9–124%)	-1.5%<br>(-47.2–44.1%)	58.9%<br>(-17.0–135%)	68.3%<br>(-115–252%)	9.4%<br>(-98.1–117%)	10.9%<br>(-50.9–72.7%)	0.72
**% Early Default**	10.2%<br>(6.8–13.7%)	9.3%<br>(0.9–17.6%)	-0.9%<br>(-5.8–4.0%)	9.4%<br>(1.2–17.5%)	9.2%<br>(-10.4–28.9%)	-0.1%<br>(-11.6–11.4%)	0.8%<br>(-5.8–7.4%)	0.80
**% Late Default**	5.6%<br>(3.1–8.1%)	3.9%<br>(-2.1–10.0%)	-1.7%<br>(-5.2–1.9%)	5.7%<br>(-0.2–11.7%)	4.5%<br>(-9.8–18.8%)	-1.2%<br>(-9.6–7.1%)	0.4%<br>(-4.4–5.3%)	0.85
**% Loss to Follow-up**	8.4%<br>(6.6–10.3%)	2.1%<br>(-2.3–6.6%)	-6.3%<br>(-8.9– -3.7%)	9.5%<br>(5.2–13.9%)	1.8%<br>(-8.7–12.3%)	-7.7%<br>(-13.9–-1.5%)	-1.4%<br>(-5.0–2.1%)	0.41

*Primary outcome for which the study was powered

Table S4.2. Comparison of change in HIV testing among males and adolescents/young adults between control and high intervention facilities after implementation of Youth Health Africa. The baseline period baseline period was January-August 2020. The study period was January-August 2021.

Proportion tested for HIV who identified as:	CONTROL (n=5) % (95% CI)			HIGH INTERVENTION (n=6) % (95% CI)			Difference-in-Difference % (95% CI)	p-value
	Baseline	Study	Difference	Baseline	Study	Difference		
**Male**	32.3% (28.1–36.6%)	31.4% (21.2–41.7%)	-0.9% (-6.9–5.1%)	31.2% (21.3–41.2%)	30.3% (6.2–54.4%)	-1.0% (-15.1–13.1%)	-0.1% (-8.2–8.0%)	0.98
**Adolescents or Young Adult**	50.4% (45.0–55.8%)	49.2% (36.1–62.3%)	-1.2% (-8.9–6.5%)	48.9% (36.2–61.7%)	49.2% (18.4–80.1%)	0.3% (-17.8–18.4%)	1.5% (-8.9–11.9%)	0.77
**Male Adolescents or Young Adult**	13.2% (10.5–15.9%)	12.4% (5.9–18.8%)	-0.8% (-4.6–3.0%)	11.9% (5.6–18.2%)	11.9% (-3.3–27.1%)	0.1% (-8.8–9.0%)	0.9% (-4.3–6.0%)	0.73

*Adolescents and young adults included ages 10-29 years old.

Figure S4. Monthly reported outcomes from high intervention and control facilities, **interrupted by intern placement in facilities in October 2020**. Points are average outcomes per month. Solid lines represent the linear model (yellow=control, blue=intervention). Dotted lines represent the 95% confidence intervals. The grey bar indicates the start of the intervention period.

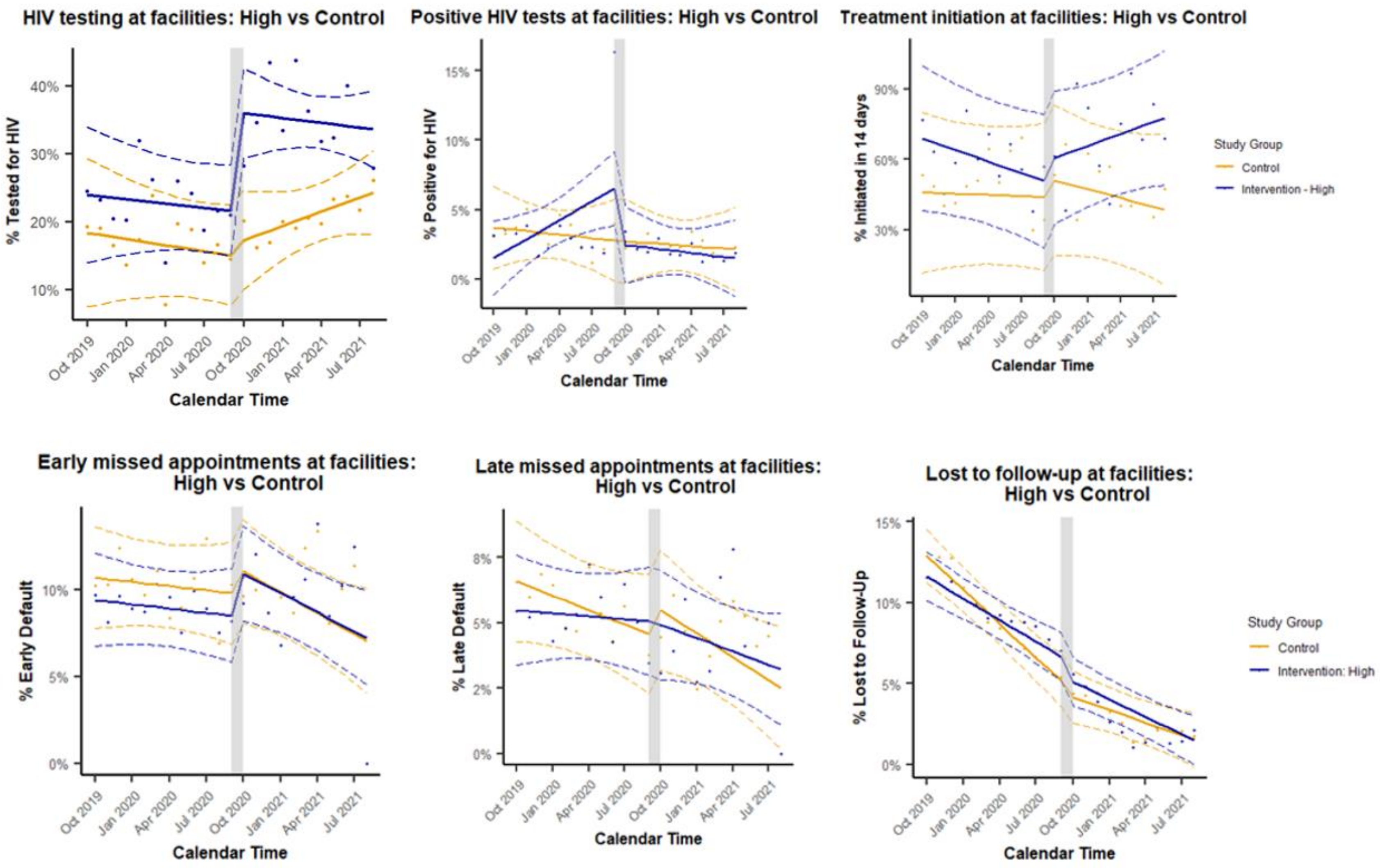

Supplement: S4 Appendix — (PDF) [file pone.0294719.s004.pdf]
