# Supplementary material for: Impact of youth lay health workers on HIV service delivery in South Africa: A pragmatic cluster randomized trial of the Youth Health Africa program
Source: PLoS One. 2023 Nov 30;18(11):e0294719. doi: 10.1371/journal.pone.0294719 (PMC10688901; doi:10.1371/journal.pone.0294719)

SUPPLEMENT 5: SENSITIVITY ANALYSIS – AS-TREATED RESULTS (LOW INTERVENTION VS CONTROL FACILITIES)

Table S5.1. Comparison of change in HIV service indicators between control and low intervention facilities after implementation of Youth Health Africa (*Difference-in-Difference analysis*). The baseline period baseline period was January-August 2020. The study period was January-August 2021.

	**CONTROL (n=5) % (95% CI)**			**LOW INTERVENTION (n=5) % (95% CI)**			**Difference-in-Difference % (95% CI)**	**P-value**
	**Baseline**	**Study**	**Difference**	**Baseline**	**Study**	**Difference**		
**% Tested for HIV***	16.5% (9.7–23.4%)	21.9% (5.3–38.4%)	5.4% (-4.3–15.1%)	18.1% (1.6–34.6%)	30.5% (-9.4–70.4%)	12.4% (-11.0–35.8%)	7.0% (-6.7–20.7%)	0.36
**% Positive for HIV**	3.0% (2.1–3.8%)	2.2% (0.2–4.1%)	-0.8% (-1.9–0.4%)	2.4% (0.3–4.6%)	1.7% (-3.4–6.9%)	-0.7% (-3.7–2.3%)	0.1% (-1.7–1.9%)	0.92
**% Initiated on Txt within 14 days**	47.6% (15.3–79.9%)	46.0% (-31.9–124%)	-1.5% (-47.2–44.1%)	61.2% (-12.1–134%)	42.6% (-134–219%)	-18.6% (-122–85.0%)	-17.1% (-77.8–43.6%)	0.56
**% Early Default**	10.2% (6.8–13.7%)	9.3% (0.9–17.6%)	-0.9% (-5.8–4.0%)	9.4% (2.5–16.3%)	9.5% (-7.2–26.3%)	0.1% (-9.7–10.0%)	1.1% (-4.7–6.8%)	0.70
**% Late Default**	5.6% (3.1–8.1%)	3.9% (-2.1–10.0%)	-1.7% (-5.2–1.9%)	5.8% (0.9–10.7%)	5.0% (-6.9–16.9%)	-0.8% (-7.8–6.2%)	0.9% (-3.2–5.0%)	0.65
**% Loss to Follow-up**	8.4% (6.6–10.3%)	2.1% (-2.3–6.6%)	-6.3% (-8.9– -3.7%)	10.9% (6.0–15.8%)	2.0% (-9.8–13.8%)	-8.9% (-15.8– -1.9%)	-2.6% (-6.6–1.5%)	0.20

*Primary outcome for which the study was powered

Table S5.2. Comparison of change in HIV testing among males and adolescents/young adults between control and low intervention facilities after implementation of Youth Health Africa. The baseline period baseline period was January-August 2020. The study period was January-August 2021.

Proportion tested for HIV who identified as:	CONTROL (n=5) % (95% CI)			LOW INTERVENTION (n=5) % (95% CI)			Difference-in-Difference % (95% CI)	p-value
	Baseline	Study	Difference	Baseline	Study	Difference		
**Male**	32.3% (28.5–36.2%)	31.4% (22.2–40.7%)	-0.9% (-6.3–4.5%)	32.2% (22.9–41.4%)	30.8% (8.5–53.0%)	-1.4% (-14.4–11.6%)	-0.5% (-8.1–7.1%)	0.89
**Adolescents or Young Adult**	50.4% (44.9–55.9%)	49.2% (36.0–62.4%)	-1.2% (-9.0–6.6%)	47.9% (34.7–61.2%)	46.5% (14.6–78.5%)	-1.4% (-20.1–17.3%)	-0.2% (-11.2–10.7%)	0.97
**Male Adolescents or Young Adult**	13.2% (10.5–15.9%)	12.4% (5.9–18.8%)	-0.8% (-4.6–3.0%)	12.4% (5.9–19.0%)	11.3% (-4.4–27.0%)	-1.2% (-10.4–8.0%)	-0.4% (-5.8–5.0%)	0.88

*Adolescents and young adults included ages 10-29 years old.

Figure S5.3. Monthly reported outcomes from low intervention and control facilities, **interrupted by intern placement in facilities in October 2020**. Points are average outcomes per month. Solid lines represent the linear model (yellow=control, blue=intervention). Dotted lines represent the 95% confidence intervals. The grey bar indicates the start of the intervention period.

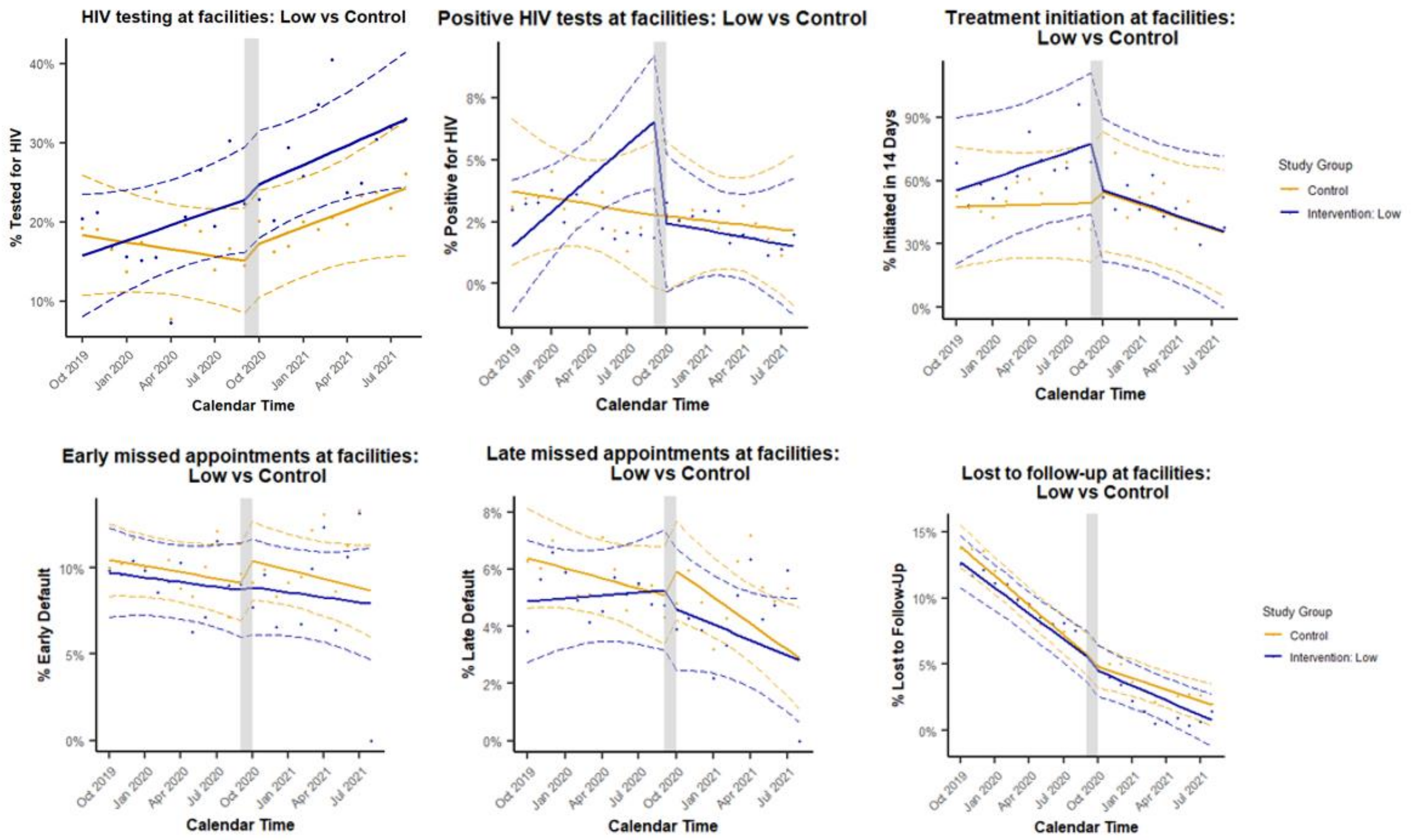

Supplement: S5 Appendix — (PDF) [file pone.0294719.s005.pdf]
