# Supplementary material for: Impact of youth lay health workers on HIV service delivery in South Africa: A pragmatic cluster randomized trial of the Youth Health Africa program
Source: PLoS One. 2023 Nov 30;18(11):e0294719. doi: 10.1371/journal.pone.0294719 (PMC10688901; doi:10.1371/journal.pone.0294719)
Supplement: S6 Appendix — (PDF) [file pone.0294719.s006.pdf]

S6 APPENDIX: SENSITIVITY ANALYSIS - % TESTED

Description and Rationale: This set of analyses excluded Facility 12, which was a "high intervention" facility, from difference-in-difference and time series analyses assessing the outcome: % Tested for HIV. This was done because a portion of monthly headcount values, which form the denominator for the outcome "% Tested for HIV" were missing and had to be imputed in the original analysis. Because this facility was a "high intervention" facility, we ran the intention-to-treat and as-treated (high vs. control) analyses for this sensitivity analysis (but not as as-treated low vs. control analysis).

For the following analyses:

Intention-to-Treat: Control facilities: n=10, Intervention facilities: n=9 intervention

As-Treated (High vs. Control): Control facilities: n=5 control; Intervention facilities: n=5

Table S6.1. Comparison of change in HIV service indicators between control and intervention facilities after implementation of Youth Health Africa (*Difference-in-Difference analysis*). The baseline period baseline period was January-August 2020. The study period was January-August 2021.

% Tested for HIV	CONTROL % (95% CI)			INTERVENTION % (95% CI)			Difference-in-Difference % (95% CI)	P-value
	Baseline	Study	Difference	Baseline	Study	Difference		
Intention to Treat	15.9% (11.1–20.7%)	21.6% (10.0–33.3%)	5.8% (-1.1–12.6%)	21.0% (9.1%-32.8%)	32.6% (4.1–61.2%)	11.7% (-5.0–28.4%)	**5.9% (-4.0–15.8%)**	0.23
As Treated: High vs Control	16.5% (9.6–23.4%)	21.9% (5.3–38.5%)	5.4% (-4.3–15.1%)	23.4% (6.8–40.0%)	35.4% (-4.6–75.5%)	12.0% (-11.4–35.5%)	**6.6% (-7.1–20.4%)**	0.32

Figures S6.2. Results of controlled interrupted time series analysis for % Tested for HIV (after exclusion of Facility 12).

Intention-to-Treat

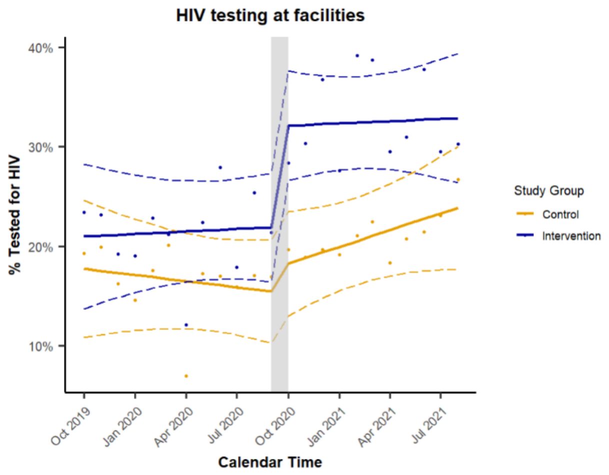

β_6Treat*Intervention: 7.9% (95% CI: 0.00-15.8%)

β_7Treat*TimeAfterIntervention: -0.8% (95% CI: -0.02-0.5%)

As-Treated (High vs. Control)

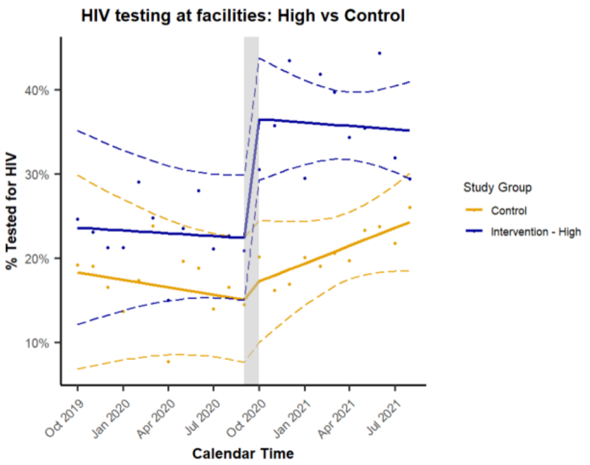

β_6Treat*Intervention: 12.7% (95% CI: 1.5-23.9%)

β_7Treat*TimeAfterIntervention: -1.0% (95% CI: -2.8-0.8%)
