# Supplementary material for: Impact of youth lay health workers on HIV service delivery in South Africa: A pragmatic cluster randomized trial of the Youth Health Africa program
Source: PLoS One. 2023 Nov 30;18(11):e0294719. doi: 10.1371/journal.pone.0294719 (PMC10688901; doi:10.1371/journal.pone.0294719)
Supplement: S7 Appendix — (PDF) [file pone.0294719.s007.pdf]

SUPPLEMENT 7: COMPARISON TO PREVIOUSLY PUBLISHED RESEARCH*

Table S7.1. Comparison of results between the interrupted time series analysis* and the cluster randomized trial.

	Time Series	Randomized Trial
**Testing**	Positive change	Initial positive increase<br><br>Overall, null (but positive signal for change)
**Testing Positive**	Null	Null
**Treatment Initiation**	Positive change	Null (but positive signal for change)
**Retention in Care**	Positive change	Null

Table S7.2. Comparison in study design between the interrupted time series analysis* and the cluster randomized trial.

	Interrupted Time Series	Randomized Trial
**Timing**	Pre-COVID<br>(Oct 2017 – March 2020)	COVID<br>(Interns placed October 2020-Aug 2021)
**Number Facilities**	207	20 (10 intervention, 10 control)
**Number Interns**	604	50 started; 34 finished
**Facility Eligibility**	▪ Aurum-affiliated<br>▪ North West or Gauteng province<br>▪ Interns started Nov 2018-Oct 2019	▪ Aurum-affiliated<br>▪ NMM district, North West<br>▪ Never had interns previously
**Intern roles & numbers**	**Varied**<br>▪ Range: 1-18 interns / facility<br>▪ Averages: 5 in Gauteng, 2 in North West	**Standardized**<br>▪ Control: 1-2 admin interns<br>▪ Intervention: 1-2 admin interns + 1-2 program interns

*Tollefson D, Dasgupta S, Setswe G, Reeves S, Churchyard G, Charalambous S, and A Duerr. “Does a Youth Intern Program Strengthen HIV Service Delivery in South Africa? An Interrupted Time-Series Analysis.” *J Int AIDS Soc*. 2023. 26(4):e26083. doi: 10.1002/jia2.26083.
