# Supplementary material for: Impact of youth lay health workers on HIV service delivery in South Africa: A pragmatic cluster randomized trial of the Youth Health Africa program
Source: PLoS One. 2023 Nov 30;18(11):e0294719. doi: 10.1371/journal.pone.0294719 (PMC10688901; doi:10.1371/journal.pone.0294719)
Supplement: S1 File — (ZIP) [file pone.0294719.s008.zip › cRCT_YHA_Data_Share_Sept18_2023/cRCT_Data_Code_Summary.docx]

**Data Sets & Analysis Code**

**Step 1: Read in Base Data Set: cRCT_base_Sept18_2023.csv**

**Step 2:** Run R file “**cRCT_finaldataprep_Sept18_2023_final.R**” to impute missing values and set up aggregate DID datasets

**Step 3**: Run DID analysis, using R file “**DID_Analysis_Sept18_2023_final.R**”

(Note: This step uses files created from Step 1)

**Step 4**: Finish setting up and run controlled interrupted time series analysis, using R file “**cRCT_cITS_Sept18_2023_final.R**”

(Note: This step uses files created from Step 1)

**Step 5**: Read in aggregate dataset: “**cRCT_age_sex_Sept18.csv**” and run DID analysis for age/sex using R file **“cRCT_DID_age_sex_Sept18_2023_final.R**”.
