# Supplementary material for: Impact of youth lay health workers on HIV service delivery in South Africa: A pragmatic cluster randomized trial of the Youth Health Africa program
Source: PLoS One. 2023 Nov 30;18(11):e0294719. doi: 10.1371/journal.pone.0294719 (PMC10688901; doi:10.1371/journal.pone.0294719)
Supplement: S3 File — (PDF) [file pone.0294719.s010.pdf]

Strengthening the South African Health System's Response to HIV through Youth Lay Health Workers:
an Impact Evaluation

Short Title: Impact Evaluation of Project Unlocked [project name changed to Youth Health Africa in 2020]

Protocol Version: 1.1 (19 October 2019)

Funder: Aurum Unlock'd Joint Venture

Implementing Partners:

The Aurum Institute for Health Research (Aurum), South Africa
Aurum Unlock'd Joint Venture [name changed to Youth Health Africa in 2020]
The University of Washington

Principal Investigator: Salome Charalambous (Aurum)

Co-Principal Investigator: Ann Duerr (University of Washington)

STUDY SUMMARY

Background: South Africa has the greatest burden of HIV globally, with numerous economic, social, and structural issues perpetuating the epidemic. Placement of disenfranchised youth as temporary lay health workers in health facilities has the potential to mitigate some of the issues that perpetuate the epidemic: shortage of human resources in the health system and youth unemployment. Project Unlocked, a Joint Venture between Aurum and Unlock'd, is training and placing disenfranchised youth as administrative and programmatic interns in health facilities in South Africa, in part to strengthen the health system's HIV response.

Aims: This study will assess whether placing youth as temporary lay health workers in clinics can strengthen the health system's HIV response in South Africa.

Objectives. This study will assess the change in health facilities' HIV program performance due to Project Unlocked. The primary objective is to examine the direct impact of the project by assessing difference in programmatic indicators related to 90-90-90 targets after nine months of implementation.

Methods: We will utilize a prospective, cluster randomized design among 18-24 facilities in Northwest Province to assess the ability of Project Unlocked to strengthen the HIV response South Africa. Facilities will be randomized to either the control arm (to receive administrative interns) or the intervention arm (to receive administrative and programmatic interns) for nine months. We will use routinely collected, programmatic data to evaluate the difference in HIV performance between control and intervention sites during nine months of implementation.

Significance: Research is needed to understand what role, if any, youth can serve as lay healthcare workers to effectively bolster the health system's HIV response. The Project Unlocked model could be applicable across the Sub-Saharan region, where countries struggle with similar issues of limited human capacity in the healthcare sector and high youth unemployment. This evaluation can therefore be used to inform scale-up of the intervention across South Africa and the broader Sub-Saharan region.

KEY PERSONNEL

**The Aurum institute**	
**Salome Charalambous (Principal investigator)**<br>Business: +27 10 590 3800<br>Cell: +27 82 856 1146<br>Email: scharalambous@auruminstitute.org	Oversees design, protocol writing, study management, study implementation, data analysis, report writing, managerial oversight and support at the sites.
**Geoff Setswe (Investigator)**<br>Email: GSetswe@auruminstitute.org	Supports study management, data analysis, and report writing
**Sibuse Ginindza (Statistician)**<br>Email: SGinindza@auruminstitutue.org	Provides statistical support for data analysis
**Aurum-Unlock'd Joint Venture**	
**Sarah Reeves (Project Manager)**<br>Email: SReeves@auruminstitute.org	Responsible for study management, study implementation, and management of sites; Supports protocol development, data analysis, and report writing
**Northwest Province**	
**Jacob Sikwane**<br>Email: JSikwane@auruminstitute.org	Supports study management, study implementation, and management of sites
**University of Washington**	
**Ann Duerr (Co-Principle Investigator)**<br>Phone: +1-206-667-7938<br>Email: aduerr@fredhutch.org	Oversees study design, protocol writing, study management, study implementation, data analysis, report writing, and managerial oversight
**Deanna Tollefson (Project Manager)**<br>Phone: +1-612-747-5573<br>Email: dtollef@uw.edu	Responsible for study design, protocol writing, study management, study implementation, data analysis, and report writing
**Sayan Dasgupta (Statistician)**<br>Email: sdg.roopkund@gmail.com	Provides statistical support for study design and analysis

Contents

STUDY SUMMARY i

KEY PERSONNEL ii

1. INTRODUCTION 1
   1.1. Background 1
   1.2. Project Unlocked 1
   1.3. Problem Statement 2

2. AIMS AND OBJECTIVES 2
   2.1. Study aims 2
   2.2. Study objectives 3
   2.3. Hypothesis 3

3. METHODS 3
   3.1. Study Design 3
   3.2. Study Location and Population 3
   3.3. Study arms 5
   3.4. Randomization of study clinics 5
   3.5. Blinding 6
   3.6. Informed consent 6
   3.7. Participant inclusion and exclusion criteria 6

4. STUDY PROCEDURES 6
   4.1. Procedures common to patients in both control and intervention arms 6
   4.2. Procedures specific to intervention arm 6

5. STUDY OUTCOME 7
   5.1. Primary Outcomes 7
   5.2. Secondary Outcomes 8

6. DATA MANAGEMENT 8
   6.1. Data Sources 8
   6.2. Data management 8

7. STATISTICAL CONSIDERATIONS 9
   7.1. Sample size estimation 9
   7.2. Statistical analyses 9

8. STUDY LIMITATIONS 10

9. PROTECTION OF HUMAN PARTICIPANTS 10
   9.1 Regulatory approvals 10
   9.2 Risks and benefits 10
   9.3 Consent 11
   9.4 Confidentiality 11

10. PROJECT GOVERNANCE AND MANAGEMENT 11
   10.1 Operations teams 11
   10.2 Management of project funds and reporting 11
   10.3 Publication policy 12
   10.4 Performance monitoring 12

11. TIMELINE 12

12. REFERENCES 13

I. INTRODUCTION

1.1. Background

South Africa has the greatest burden of HIV globally, home to one-in-five people infected with HIV, or approximately 7.7 million people living with HIV (PLHIV) (1). While South Africa has made great strides in addressing the HIV epidemic, much work remains to reduce new infections and reach the level of detection, treatment, and viral suppression necessary to quell the spread of HIV (1,2). In 2018 alone, an estimated 240,000 people were newly infected with HIV in South Africa (1). Furthermore, it is estimated that while 90% of PLHIV know their status, only 62% of these are on treatment and only 54% have reduced viral loads (1)— numbers that fall far short of the 90-90-90 targets that must be met to reverse the HIV epidemic (2). Youth, especially young women, bear a disproportionate burden of HIV (3), and they lag behind other age groups for testing and treatment (4,5).

Numerous underlying economic, social, and structural issues perpetuate the epidemic in South Africa (6). In particular, shortages of human resources have resulted in a significant challenge for the public healthcare system that has impeded the HIV response (7), as insufficient human capacity can hinder quality of and linkage to care (8). Improved clinic operations have been linked to improved care, e.g., uptake of ART (9), but are difficult to undertake with limited human resources. Economic pressures can also drive the HIV epidemic. Disenfranchised youth, i.e., adolescents and young adults with limited access to education and employment, are especially at risk for HIV (10–12). Addressing this issue is imperative to ending HIV in South Africa where over half of people aged 15-24, and 38% of people aged 15-35, are unemployed (13).

These challenges are not unique to South Africa but are rather ubiquitous across Sub-Saharan Africa. Health systems across low and middle-income countries struggle from inadequate human resources (14), and youth unemployment is one of the biggest development challenges for many Sub-Saharan African countries (15,16).

Disenfranchised youth represent a wealth of potential for the understaffed health system in South Africa. Lay healthcare workers, paid or unpaid, have been viewed as a critical piece of strengthening linkage to healthcare in many settings (17). Past research shows that lay health workers can significantly improve linkage to care, but numerous challenges exist in ensuring quality and sustainable lay health worker interventions (18–23). In particular, there is little known on whether placing disenfranchised youth as lay healthcare workers could bolster the health system's HIV response. Research is therefore needed to understand what role, if any, youth can serve as lay healthcare workers to effectively bolster the HIV response in South Africa. The findings of such research could be applicable not just in South Africa, but across the Sub-Saharan region, where countries struggle with similar issues of limited human capacity in the healthcare sector and high youth unemployment.

1.2. Project Unlocked

In South Africa, a joint venture between Aurum Institute and Unlock'd[1], a local youth talent development business, was launched in 2018 to roll-out a novel youth unemployment project designed to increase the employability of disenfranchised youth by training and placing youth in one-year internships at health facilities. (The Joint Venture between Aurum and Unlock'd is henceforth called Project Unlocked in this protocol.) This project has dual goals to (1) improve the employability and livelihoods of the youth it engages, but also (2) strengthen the HIV response in South Africa. Youth are placed in HIV-related programmatic and administrative roles in health clinics; they receive training and mentorship from program leaders as well as a designated supervisor at the facility so they can be successful on the job.

While the youth learn skills that will make them more employable post-internship, the youth are also contributing to the HIV response of the health facility where they are placed. Youth, who are most commonly referred to as "interns" in Project Unlocked, can be placed in a wide number of roles, ranging from

[1]More on the Unlock'd organization can be found at http://www.unlockd.co.za/site/index .

administrative (e.g., file clerks) to programmatic (e.g., testing people for HIV or helping people who have tested positive for HIV to navigate care). In many cases, interns fill roles that were previously vacant. In other cases, interns fill new positions that the facility created to bolster programmatic or administrative support to clinic staff. Examples of the roles that youth occupy are outlined below in Figure 1.

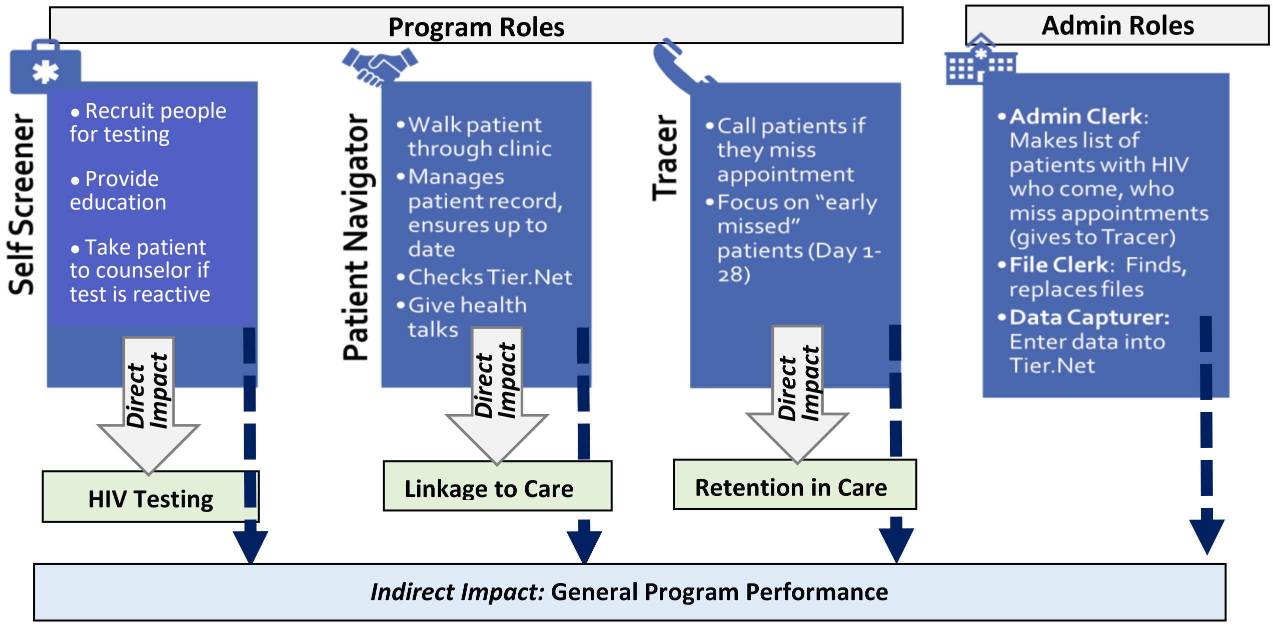

Figure 1. Overview of the programmatic and administrative roles that interns fill in Project Unlocked, and the potential to impact HIV programs in the health systems where they are placed.

1.3. Problem Statement

While Project Unlocked has scaled up rapidly in its first year of operation, there is a need to conduct a rigorous evaluation of the project to determine its impact on the health system. While many programs use lay health workers for health system strengthening, none to our knowledge specifically rely on identifying and training unemployed youth, specifically youth who are themselves at greater risk of HIV, to fill these roles on a temporary basis. While the Joint Venture has preliminary evidence to suggest that Program Unlocked benefits the interns, evidence is needed to understand the impact the program has on the health system in which it operates.

The proposed study will therefore assess whether placing youth as temporary lay health workers in health facilities can strengthen the health system's HIV response in South Africa. We will utilize a nine-month prospective, cluster randomized design to study the ability of this novel youth employment project to strengthen the HIV response in South Africa as implemented under routine, programmatic settings.

2. AIMS AND OBJECTIVES

2.1. Study aims

This study seeks to determine the impact that temporary youth interns, who serve as lay healthcare workers through Project Unlocked, have on the health system's HIV program performance. Specifically, we aim to understand what impact, if any, Project Unlocked interns have on the ability of the health facilities where they are placed to test people for HIV, initiate newly diagnosed HIV-positive persons on treatment, and retain people with HIV in care, as we hypothesize these are the portions of the program that the interns can directly impact (see Figure 1). That is, interns serving as self-screeners would be expected to impact HIV testing rates, while interns serving as patient navigators would be expected to impact linkage to care, and interns serving as tracers would be expected to impact overall retention in care. There could also be broader effects on the HIV program

(e.g., TB case finding, initiation of TB prophylactic treatment). A controlled study is needed to ascertain what, if any, impact this project has on these indicators.

Assessment of these aims involves analysis of routinely collected facility data (e.g., data Aurum receives to monitor facility performance and report to PEPFAR). **No primary data collection will be required**. Study objectives will be studied through use of routinely collected data, which Aurum routinely uses to monitor clinics (e.g., TIER.Net and DHIS).

2.2. Study objectives

Primary Objective: The primary objective of this study is **to quantify the change** in HIV program performance among health facilities with Project Unlocked interns in programmatic roles (intervention sites) versus health facilities where Project Unlocked interns are not in programmatic roles (control sites) by examining the difference in programmatic indicators related to **90-90-90 targets**.

- **HIV testing (90)**: Percentage of individuals who received HIV testing services (HTS) and received their results, among all persons who were seen at the facility.
- **Linkage to treatment (90-90)**: Percentage of individuals testing positive who were linked to treatment at the facility (same day of testing and by 14 days post-testing).
- **Retention in care (90-90-90):** Percentage of people on treatment for HIV who default from treatment. This measure will be used as a proxy for viral suppression, which is the "third 90" of the 90-90-90 targets.

Analyzing the 90-90-90 targets is the primary objective for this study, as the interns in programmatic roles are performing activities directly linked to these indicators, i.e., testing, linkage to care, and retention in care (see Figure 1). As such, we have reason to believe interns could **directly impact performance**, as measured by these indicators.

Secondary Objective: We will measure how the presence of interns at health facilities **indirectly** impacts other HIV services that are not directly part of the programmatic interns' work responsibilities, such as initiation and completion of isoniazid preventive therapy (IPT) among people newly diagnosed with HIV. The indicators used to measure the secondary objectives will also be those routinely reported (i.e., through DHIS and TIER.Net).

2.3. Hypothesis

We hypothesize that intervention clinics will experience a significant improvement in HIV testing, linkage to care, and retention in care as compared to control clinics, as program interns will directly contribute to improvements in related services. We hypothesize that intervention clinics will also experience improvement in other HIV programmatic areas that are not directly related to the roles and responsibilities the interns hold.

3. METHODS

3.1. Study Design

We propose a nine-month cluster-randomized controlled trial (cRCT) with 18-24 clinics in Northwest Province to quantify the impact of Project Unlocked on HIV testing, treatment, and retention in care. Health facilities, and not individuals within the facilities, will be the unit of analysis. No data from individuals will be collected. All data will be collected at the facility level. Only routinely collected data will be utilized in this study.

3.2. Study Location and Population

HIV facilities: This study will primarily be conducted among health facilities in Ngaka Modiri Molema (NMM) district in Northwest Province, for which Aurum is the PEPFAR implementing partner. We selected NMM district because the majority of facilities in this district have not yet participated in Project Unlocked but have expressed interest in the program. There 31 facilities in NMM district currently eligible for this study. These facilities and accompanying details are described in Table 1 (see next page). Project activities will be expanded to facilities in

other districts in Northwest Province if needed for operational reasons or to maintain the power required for this study.

HIV clinics are actively recruited to participate in Project Unlocked. Aurum uses its position as Implementing Partner to identify clinics where additional capacity for non-clinical HIV activities is necessary, and thus identify clinics that could benefit from the Project Unlocked model.

Youth Interns: Project Unlocked passively recruits youth to participate in this project, relying on flyers at community-level health facilities and word of mouth to encourage application to the program. The target youth population for the project is aged 18-30 with low socioeconomic status, minimal education, and limited job experience. The median age of participation is approximately 25 years; youth under 18 are not part of this project. Youth are selected into the program based on the level of need at the HIV clinics in their communities, not based on the youth's skill set or experience.

Table 1. Facilities in NMM district, Northwest Province eligible for inclusion in proposed cluster RCT. Averages were calculated from October 2018-July 2019.

Facility name	Local Municipality	Avg No. Patients / Month	Avg No. Tested/ Month	Avg No. Newly Positive/ Month	Avg No. Initiated/ month	Avg No. Initiated on same day/ month	Total No. On ART (July 2019)	Total No. Default Early (July 2019)
**Agisanang Clinic**	Tswaing	1833	264	7	7	4	740	76
**Bakerville Clinic**	Ditsobotla	1075	72	3	4	1	327	67
**Blydeville Clinic**	Ditsobotla	1244	116	9	8	4	577	99
**Blydeville Old**	Ditsobotla	1640	209	12	10	5	719	139
**Bodibe 1 Clinic**	Ditsobotla	3274	501	14	14	10	1181	225
**Boikhutso Clinic**	Ditsobotla	3907	669	21	20	10	1577	268
**Braklaagte**	Ramotshere Moiloa	1661	105	5	5	4	536	72
**Coligny CHC**	Ditsobotla	3033	426	14	14	2	1013	200
**Delareyville CHC**	Tswaing	2938	416	23	24	17	1331	335
**Dinokana CHC**	Ramotshere Moiloa	3593	453	12	14	10	1211	104
**Dinokana old**	Ramotshere Moiloa	1528	184	6	6	2	702	43
**Driefontein**	Ramotshere Moiloa	837	85	1	2	1	249	38
**Ganalaagte Clinic**	Tswaing	2948	244	10	13	6	1254	189
**Gopane**	Ramotshere Moiloa	1882	226	4	4	0	501	45
**Groot Marico**	Ramotshere Moiloa	1072	147	6	5	3	494	92
**Itekeng Clinic**	Ditsobotla	1419	205	4	5	4	526	137
**Itsoseng CHC**	Ditsobotla	4350	469	13	13	4	1279	191
**Itsoseng Clinic**	Ditsobotla	1555	105	5	5	2	537	105
**Kunana Clinic**	Tswaing	1949	141	4	5	0	581	82
**Lehurutshe Hospital**	Ramotshere Moiloa	1462	184	7	8	1	181	24
**Letsopa Clinic**	Tswaing	2885	447	12	14	7	966	124
**Mokgola**	Ramotshere Moiloa	1611	159	4	5	3	486	57
**Motswedi**	Ramotshere Moiloa	1344	221	5	5	3	485	44
**Ntsweletsoku**	Ramotshere Moiloa	1034	20	3	6	1	425	86
**Ottosdal CHC**	Tswaing	2892	255	10	9	4	828	335
**Rietpan**	Ramotshere Moiloa	750	134	3	4	2	243	18
**Sannieshof CHC**	Tswaing	1550	97	7	6	3	449	59
**Tlhabologang Clinic**	Ditsobotla	2475	289	9	9	4	680	211
**Tswelelopele CHC**	Ramotshere Moiloa	2698	394	13	12	7	1040	98
**Vriesgewacht Clinic**	Tswaing	1598	145	4	5	3	447	79
**Zeerust**	Ramotshere Moiloa	1272	271	14	11	10	736	56

3.3. Study arms

There will be an equal number of facilities in the control and intervention arms (1:1 randomization). Facilities are the unit of analysis and will be randomized to the control or intervention arms. Each participating facility will receive a similar number and allocation of youth interns at the facility for a year.

Control arm: Facilities in the control arm will receive the minimum package for the intervention at baseline, i.e., 1-2 interns who will be assigned to administrative roles, such as admin clerk, file clerk, or data capturer.

Intervention arm: Facilities in the intervention arm will receive the minimum package *and* the intervention package at baseline. That is, they will receive 1-2 interns placed in administrative roles (admin clerk, file clerk, or data capturer) and 2-3 interns placed in programmatic roles according to the needs of the facility, one of each of the following: self-screener, patient navigator, and/or tracer. The number of interns allocated to the clinic will be based on the size of the clinic; whether the clinic receives two or three program interns, they will cover self-screening, patient navigating, and tracing. Interns engaged in self-screening will provide pre-counselling to patients and support the patient's use of the self-screening kit. The intern will provide a post-counselling session if the patient tests negative or connect them with one of the facility's lay counsellors if the test is positive. (Note: The self-screening test is used only as a screening test and requires confirmation using traditional testing methods if positive, per South Africa's current national protocol.) Interns placed as patient navigators and tracing will support roles as outlined in Figure 1 (see page 2).

We will aim for control and intervention sites will be similar in all essential regards, other than receipt of the intervention package, as all facilities come from a similar geographic region (i.e., NMM district, Northwest province, South Africa) and are similarly managed (i.e., Aurum is the implementing PEPFAR partner for HIV work at each facility). While differences between facilities do exist, randomization of facilities to the control or intervention arms will result in balanced differences between arms and minimize bias. Preliminary data collection at clinics will be done prior to randomization and restriction may be applied based on those results.

3.4. Randomization of study clinics

All facilities that are eligible and willing to participate in this study (18-24 facilities) will be randomized to the control or intervention arm. We will use stratification improve similarity between facilities in the treatment and control groups. Facilities will be, at minimum, stratified by HIV programmatic performance (measured by HIV testing) and, if necessary, also stratified by size (measured by total monthly head count at the facility). The final decisions for stratification will be driven by the facility data observed in the year proceeding the study. The minimal proposed stratification is outlined in Figure 2.

We will randomize an equal number of facilities to the control or intervention group within each strata using a random number generator. We will randomly assign facilities at a joint meeting between study implementors and program staff (e.g., the NMM health district manager).

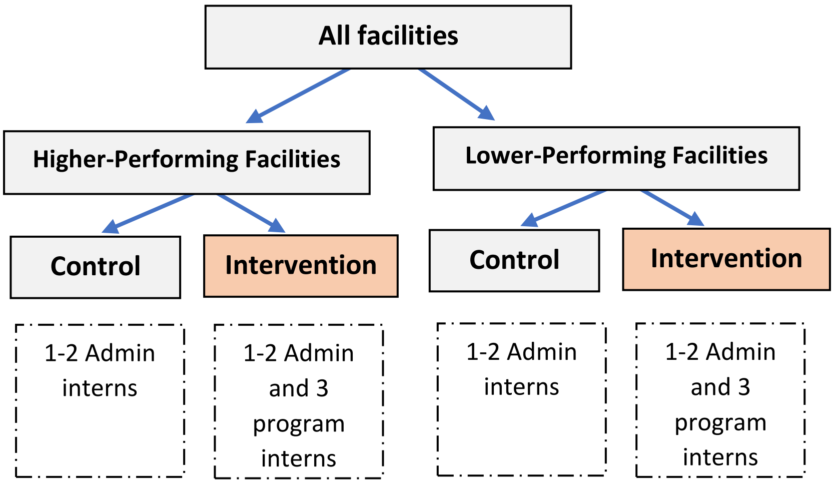

Figure 2. Allocation of facilities to control or intervention

3.5. Blinding

There will be no blinding in this study. Randomization will be conducted publicly with all clinic managers and district officials will be invited. Cooperation with program leaders is essential to maximize facility retention in the study.

3.6. Informed consent

Facilities are the subject and unit of analysis in this study. Project Unlocked implementers will approach facilities with an overview of the study and invite them to participate, after having received permission from the Northwest Department of Health. Facilities are free to decline the invitation to participate without repercussion. Facilities interested in participating will be asked for verbal agreement to engage in the study, but this study does not require consent.

No consent is required for this study from either the individual or facility level, as all activities are programmatic in nature and the study involves no data collection (i.e., there will be no primary data collection, as only routinely collected data will be used for the analysis). This is an evaluation of a routinely implemented program. No individuals will be randomized and no individual-level data will be collected for this study. Only aggregate-level program data will be used for the evaluation.

3.7. Participant inclusion and exclusion criteria

Inclusion: Facilities must be in the Northwest Province with Aurum as the PEPFAR implementing partner to be eligible to participate. To be eligible, facilities must have a demonstrated need for administrative and programmatic interns. Facilities must also express a willingness and interest to engaged with Project Unlocked. They must be willing and interested to receive an entire package of interns that fill both administrative and programmatic roles. For facilities to be considered eligible for this study, they must not have previously received interns, other than for administrative support, from Project Unlocked.

Exclusion: Facilities are ineligible if they are not interested in receiving both administrative and programmatic interns, and if they have no demonstrated need for these interns (e.g., the facility already has people placed in roles of self-testers, patient navigator, and patient tracer).

4. STUDY PROCEDURES

4.1. Procedures common to patients in both control and intervention arms

All facilities in this study will retain their normal staff and receive the support of a minimum package of administrative interns from Project Unlocked. The minimum package consists of 1-2 interns that will fill roles of admin clerk, file clerk, and/or data capturer. It is important that all participating facilities receive this minimum package to ensure similar data quality between the intervention and control groups.

The analysis for this evaluation utilizes routinely collected data. We understand that the addition of staff who serve in administrative roles, such as data capturers or file clerks, can impact the quality of data. To understand the impact that interns have on HIV services, it is imperative that both the control and treatment facilities have a similar level of data quality. As such, both the treatment and control facilities will receive this minimum package of administrative interns. Aurum will provide additional support to supervise data capture at both intervention and control sites, if it is necessary to ensure data are being routinely captured at all sites.

Ensuring the control and intervention group both receive a minimal package of interns will also serve to increase willingness to participate in this study, as all facilities will be assured a base number of interns at baseline.

4.2. Procedures specific to intervention arm

The intervention arm will receive an intervention package that consists of 2-3 interns assigned to the roles of self-screener, patient navigator, and patient tracer. These roles are directly responsible for HIV testing, linkage

to care, and retention in care, respectively (see Figure 1). The exact number of interns at an intervention facility may differ based on the size and needs of the facility. However, the interns placed at the facility will cover these three roles, with one intern being able to occupy more than one role, if necessary.

At nine months the study will conclude, at which time the control facilities will also be offered the treatment package (i.e., 2-3 programmatic interns). The difference in staff between the control and treatment facility is summarized in Table 2 below.

Table 2. Comparison of procedures between control and treatment facilities.

Control Facility	Treatment Facility
**Routine Staff** + **Administrative Interns** (File clerk, admin clerk, and/or data capturer)	**Routine Staff** + **Administrative Interns** (File clerk, admin clerk, and/or data capturer) + **Program interns** covering the roles of self-screener, patient navigator, and patient tracer

5. STUDY OUTCOME

5.1. Primary Outcomes

There are three primary outcomes of interest in this study, which will be assessed for a total of **six months** through a combination of variables that reported monthly through TIER.net to Aurum. The indicators that will be used to assess the primary outcomes of interest are summarized in Table 3 below.

First Outcome: % **Tested for HIV** –*Number of individuals who received HIV testing services (HTS) and received their results out of all individuals visiting the clinic for services.* This will be calculated through use of two standard indicators.

- % Tested for HIV = HTS_TST / Head Count

Second Outcome: % Linked to Treatment –*Number of individuals testing positive who were linked to treatment at the facility, out of all individuals who tested positive for HIV.* There will be two measures we examine:

- % Linked to care on day of testing = Initiated Same Day / HTS_TST_Pos
- % Linked to care within 14 days of testing = Initiated 14 Days / HTS_TST_Pos

Third Outcome: % Retained in care –*Number and percentage of people on treatment for HIV who default from treatment (early default, late default).* There will be two measures we examine:

- % Early Default = ART Default Early / Total on Treatment
- % Default = ART Default Late / Total on Treatment

We will rely on indicators reported through routine reporting mechanisms, primarily relying on TIER.Net due to its monthly frequency of reporting and the high number of indicators included in these reports. The numerators and denominators for each indicator will come from the same reporting period (i.e., the same month).

While the full study is nine months in duration, the first three months will be a "run in" period, data from which will not be used to assess study outcomes. We will use data from months 4-9 (i.e., six months of data) of the intervention period to calculate these outcome measures.

Table 3. Indicators that will be used to measure outcomes of interest are reported monthly through TIER.Net.

Outcome	Indicator	Indicator Definition
% Tested for HIV	Head Count	Number of individuals visiting the clinic for services
	HTS_TST	Number of individuals who were tested for HIV
% Linked to Treatment	HTS_TST_POS	Number of individuals who tested positive for HIV
	Initiated Same Day	Number of individuals who were started on ART on the same day they tested positive for HIV
	Initiated 14 Days	Number of individuals who were started on ART within 14 days of testing positive for HIV
% Retained in Care	TX_Curr	Number of individuals currently receiving ART at the facility
	ART Default Early	Number of individuals who missed treatment within the first 28 days after initiation
	ART Default Late	Number of individuals who missed treatment after 28 days from date of initiation

5.2. Secondary Outcomes

In addition to the indicators in the table above, we will examine other programmatic indicators available in TIER.Net, namely IPT initiation and completion, to sense possible indirect effects Project Unlocked has on the HIV programmatic services offered at the engaged facilities.

6. DATA MANAGEMENT

6.1. Data Sources

This study does not involve primary data collection. The study will utilize routinely collected programmatic data aggregated at the facility level for analysis. No additional primary data will be collected. No patient-level data will be accessed or otherwise used in this analysis. As such, there is no Personal Identifiable Information (PII) that will be included in this dataset.

Existing facility-level data will be abstracted from routine programmatic reports provided to Aurum, e.g., Quality Insurance (QI) reports reported on a monthly basis from TIER.Net. Data from these reports will be used to review study-associated outcomes to evaluate the impact of Project Unlocked on health facilities' HIV programs. To achieve this, data on key HIV indicators collected on a monthly basis from baseline (0 months) to 9 months post-intervention. Indicators will be compared between intervention and control facilities at key months (see Section 5). The key indicators that will be used in this analysis are described elsewhere (Section 5 and Table 3).

6.2. Data management

Monthly facility-level data for 10 months (baseline through 9 months) of key indicators along with facility-level characteristics at baseline (i.e., sub-district, facility size, treatment group), will be collated into a CSV file. Data will be abstracted from the routinely reported monthly, Excel-based reports into this dataset. After all data have been merged, the baseline dataset will be frozen and designated members of the study team will conduct data cleaning in R.

Structure of Dataset

The dataset will include one sheet and be in wide format (i.e., one row per facility). There will be one column per indicator of interest per month. There will be one row per facility, to enable easy aggregation of data collected per facility from months 4-9 of the intervention period. Data will be cleaned and organized in R.

Data entry validation

All data entry will be conducted under routine programmatic conditions. Program staff will use routine, existing facility-level protocols to enter and submit their data to Aurum and other entities. This study will not propose variations on this routine process (i.e., there is no data entry validation specific to this study). While one study member will collate the dataset from monthly reports, a secondary member will review the dataset to ensure quality of the final dataset. The dataset will be collated after the intervention period (i.e., at month 9). This will ensure that the most up-to-date data are captured in our analysis, and that the study team does not use the data to prospectively alter performance of the intervention.

Data cleaning

Study staff will review the routinely reported data for outliers and send inquiry to program staff on any outliers. Study staff will adjust any outliers in consultation with the facilities that generated the data.

Access to dataset

The dataset will be password protected and shared only with study and program staff.

Locking of database

The dataset will be cleaned in a standard statistical program (e.g., STATA or R). The dataset will be locked after all data have been merged and reviewed for outliers.

Information security

The dataset will be password protected and shared with the University of Washington through Aspera data transfer, a protected web portal for data sharing. All data are deidentified, containing only aggregated numbers at the facility level. Access to the database (data entry, reporting, and extraction) will be controlled by the study team. Study personnel requiring access to the database must will complete any required documentation and training required by Aurum prior to receiving access to the data.

Data ownership

All data will be owned by Aurum, but researchers with University of Washington will be primarily responsible for analysis, and thus will have access to it for cleaning and analytic purposes.

7. STATISTICAL CONSIDERATIONS

7.1. Sample size estimation

Sample size: We will enroll 18-24 clinics in this study, resulting in 9-12 clinics being in the treatment and the control arms of the study. The sample size is pre-determined by the number of available eligible clinics.

Study power: In Table 4 below, we show the minimum detectable differences in HIV testing that can be detected with 80% power under different assumptions of the intra class correlation (ICC) (0.01-0.05), at 5% level of significance, and for different cluster size (number of facilities). For example, if there are 24 facilities in this study (12 in each of the study arms), we will have 80% power to detect a difference of at least 35% in HIV testing when the ICC is 0.01, and at 5% level of significance. Similarly, if we have 18 facilities in this study (9 in each of the study arms), we will have 80% power to detect a difference in HIV testing of at least 45% when the ICC is 0.01, and at 5% level of significance.

These power calculations are based on data on the eligible facilities from the NMM district that show there was an average of 1,687 people tested for HIV in six months per clinic and that average HIV testing was at 14% in this time period.

Table 4. Assumptions for power calculations, with estimated HIV testing level needed to achieve 80% power.

Significance level	Power	Current proportion of total headcount tested for HIV	Number of clusters	Average cluster size	Coefficient of variation (cv)	ICC	Proportion of headcount tested for HIV needed to achieve 80% power
5%	80%	0.14	12	1687	0.596	0.01	0.19
						0.03	0.23
						0.05	0.26
5%	80%	0.14	9	1687	0.596	0.01	0.20
						0.03	0.25
						0.05	0.28

Statistical analyses

The first three months of the study comprise the “run in” period. Data from the wash-out period will not be used in the analysis. We will use six months of facility data collected during months 4-9 of the intervention for this analysis.

We will employ ‘Cluster-level’ methods to conduct statistical analysis in a two-stage approach here. The first step will consist of estimating a summary measure for each cluster (facility): we will calculate the % tested for

HIV, % linked to treatment, and % retained in care in each facility for the six months of the intervention period. We will calculate this by aggregating each indicator, then calculating the outcomes of interest (percentages) based on the six-month total counts. In the second step, we will carry out weighted 2-sample t-tests to test the null hypothesis that there is no significant difference between the intervention and the control groups with respect to these summary measures. Weighted approaches are preferred here, as they have been shown to improve efficiency when clusters are not of the same size [24], which is the case in our trial. In our approach, the weights are set to be the inverse of the estimated variance of the cluster summary measures, that is,

$$w_i = \frac{1}{v_i + \sigma^2}$$

where v_i is the variance of the cluster level summary for facility i and σ^2 is the between-cluster (between-facility) variance. We will also conduct additional analyses adjusting for facility-level factors as necessary.

Should changes in testing, linkage to care, and retention in treatment be noted in the analysis, we will descriptively examine these indicators by sex and age groups of interest to understand what demographic was receiving additional testing and/or care as a result of this program. These indicators are available in aggregate in the routinely reported data.

8. STUDY LIMITATIONS

While this study has numerous strengths, namely its cluster randomized design, it has limitations that must be acknowledged.

- **Use of routinely collected data** – We will be use routinely collected data from TIER.Net for our analysis. While this has many advantages (e.g., no additional resources are needed to collect this data), its quality can be variable based on capacity at the clinic to enter the data. The addition of administrative interns into both the control and the treatment site clinics will help ensure strong quality data at all sites involved in the study.
- **Limited duration of follow-up** – To ensure that the control sites can receive program interns in a timely fashion, the duration of this study (nine months) is shorter than ideal. Having a longer follow-up period would increase the likelihood of observing impact due to the intervention, but this would be unfair to the control sites who could also potentially benefit from the support of program interns. A short follow-up period could result in us missing a true effect because there was not enough time for it to materialize. However, a nine-month study period will ensure high facility participation and retention in the study.
- **Routine programmatic conditions** – This cluster randomized trial is being conducted under routine program conditions. This means that while we will do our best to encourage facilities to retain interns in their designated roles, facilities can place interns in the roles and capacities of their choosing and the intervention may not be implemented with fidelity.
- **Small size** – This study will include 18-24 clinics, distributed equally among the control and intervention arms. As the facility is the unity of analysis, this yields approximately 9-12 units per study arm. This limitation is unavoidable, as this is the maximum number of clinics that can participate in the study due to eligibility and resource constraints.

9. PROTECTION OF HUMAN PARTICIPANTS

9.1 Regulatory approvals

Ethical approval for the study will be sought from the University of Witwatersrand Research Ethics committee and the University of Washington. We will also seek permission for conduct of the study from the Research Committee of the North West province.

9.2 Risks and benefits

Risk is minimal in this study. The primary risk is that the interns assigned to clinic work could be insufficiently trained and thus detract from routine facility operations. However, Project Unlocked has not noticed such

adverse consequences to its intern program to date, thus obvious negative consequences are not anticipated. Secondly, the evaluation could reveal less than optimal implementation strategies or results from Project Unlocked. Any negative findings will be passed on to project leadership who can use these to improve the project. Finally, the delay in assignment of program interns to facilities in the control arm could delay progress the facilities may have observed if interns were placed there earlier. However, delayed receipt of interns is preferable than the status quo of no interns. All data for this study is routinely collected and aggregated, thus there is no risk of privacy to HIV-infected patients.

The primary benefit to this study is the addition of resources to all participating clinics. All facilities are receiving at minimum administrative interns that could directly and/or indirectly increase clinic capacity to manage patients. This could enable facilities to do more HIV testing, link people who tested positive to care faster, and increase retention in care, amongst other things. Indirectly, the extra human capacity could reduce workload on nurses currently working at the clinics, thus enable nurses to better engage with their patients. Moreover, the evaluation could identify ways in which the project could improve, thereby having a bigger impact in the long term.

9.2 Consent

There will be no individual-level data included in this analysis. We are not collecting data on interns, negating the need for consent from these individuals. At the program level, all interns participate in the internship of their own volition and are able to resign from their role at any point in time, although their position is guaranteed for a year. All interns participating in this program are young adults (at least 18 years old).

Facilities are the subject and unit of analysis in this study. Eligible facilities will be invited to participate in the intervention and can decline without consequence. Leaders at the facilities interested in participating will verbally agree to engage in the study, but no consent is necessary as this study is a program evaluation relying solely on routinely collected program data for its analysis.

9.3 Confidentiality

Deidentified, facility-level data will be sent to the study team for analysis. This study does not involve primary data collection. There will be no paper or electronic records specific to this study that have not already been collected for routine programmatic reporting and management. We will ensure that the study dataset, which is comprised of this programmatic data, is managed in a secure and confidential fashion. Access to the records will be restricted to specified study team members and the dataset will be password protected.

10. PROJECT GOVERNANCE AND MANAGEMENT

10.1 Operations teams

This study team is ideally situated to complete the proposed research due to close working relationships with the province and the implementing facilities. The Principal Investigators will oversee the project. The Project Managers will be responsible for planning the intervention and monitoring of the intervention. University of Washington will be responsible for data analysis and will lead production of written report and manuscripts.

10.2 Management of project funds and reporting

This evaluation does not require specific funding (i.e., it does not require specific field visits or data collection). Personnel who are already funded will provide the management and analytic support for this project.
The intervention will be implemented with routine Project Unlocked funds, just as it is implemented in non-study settings. General oversight of these funds is provided through routine project management channels in the Aurum Unlock'd Joint Venture that are outside the scope of this study. General oversight for the project will

be provided by the Principal Investigator who will receive regular updates from investigators on the Aurum Unlock'd Joint Venture team.

10.3 Publication policy

The research findings will be presented first to provincial stakeholders through local meetings, and then to national stakeholders, as relevant. The results of this evaluation will be written up in one or more articles for submission to a suitable peer-reviewed journal and submitted to pertinent national or international conferences. A Policy Brief will also be drawn up for dissemination to the National Department of Health.

10.4 Performance monitoring

The investigator team will meet every two weeks to monitor performance of the study, with Sarah Reeves (Aurum Unlock'd Joint Venture) and Deanna Tollefson (University of Washington) providing study updates on vital information, such as IRB timelines, status of protocol development, enrolment figures, and any issues/delays that the study might be experiencing.

11. TIMELINE

From start to finish, this study will last approximately two years, with the active study period occurring over nine months (Table 5). Study design and enrollment will occur in latter 2019. The intervention will be implemented from December 2019 through August 2020 (nine months). The first three months of the study period will be considered a run-in period, with the understanding that it will take time for the interns to be trained and fully integrated into the facilities where they work. Months 4-9 (six months) will be considered the "intervention period" and will be used in the analysis. This is depicted in Figure 3 below. Data cleaning and analysis will occur in Quarter 4 of 2020. Results will be ready for dissemination by mid-2021.

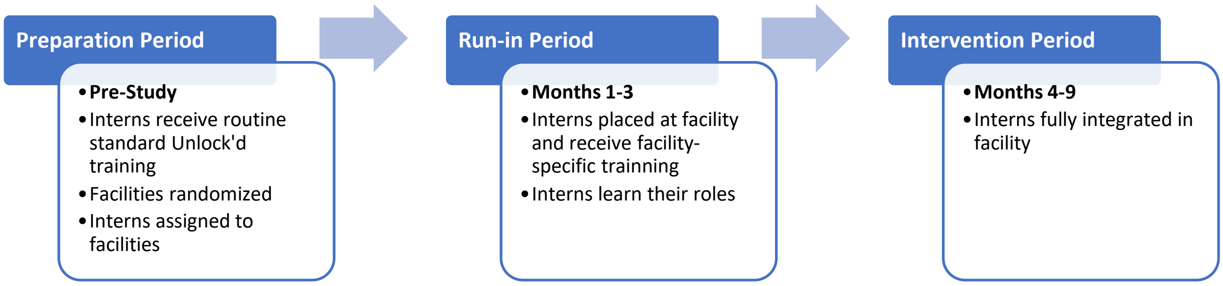

Figure 4. Time period for intervention and study period.

Table 5. GANTT chart for proposed study

	2019		2020				2021	
	Q3	Q4	Q1	Q2	Q3	Q4	Q1	Q2
**Study Planning**								
Protocol Development	X							
Community Engagement	X							
Enrollment		X						
Randomization		X						
**Implementation**								
Learner training		X						
Run-in period		X	X					
Data collection			X	X	X			
**Analysis**								
Data cleaning						X		
Data analysis						X		
**Dissemination**								
Write up							X	
Disseminate results								X

12. REFERENCES

1. South Africa | UNAIDS [Internet]. [cited 2019 August 29]. Available from: http://www.unaids.org/en/regionscountries/countries/southafrica

2. UNAIDS. Miles to go—closing gaps, breaking barriers, righting injustices | UNAIDS [Internet]. 2018 [cited 2019 May 2]. Available from: http://www.unaids.org/en/resources/documents/2018/global-aids-update

3. Simelela N, Venter WDF, Pillay Y, Barron P. A Political and Social History of HIV in South Africa. *Curr HIV/AIDS Rep*. 2015 Jun;12(2):256–61. Available from: http://www.ncbi.nlm.nih.gov/pubmed/25929959

4. Zgambo M, Kalembo FW, Mbakaya BC. Risky behaviours and their correlates among adolescents living with HIV in sub-Saharan Africa: a systematic review. *Reprod Health*. 2018 Dec;15(1):180. Available from: http://www.ncbi.nlm.nih.gov/pubmed/30355344

5. Adejumo OA, Malee KM, Ryscavage P, Hunter SJ, Taiwo BO. Contemporary issues on the epidemiology and antiretroviral adherence of HIV-infected adolescents in sub-Saharan Africa: a narrative review. *J Int AIDS Soc*. 2015 Jan;18(1):20049. Available from: http://www.ncbi.nlm.nih.gov/pubmed/26385853

6. Fox AM. The social determinants of HIV serostatus in sub-Saharan Africa: an inverse relationship between poverty and HIV? *Public Health Rep*. 2010;125 Suppl 4(Suppl 4):16–24. Available from: http://www.ncbi.nlm.nih.gov/pubmed/20629252

7. Rispel L, Blaauw D, Ditlopo P, White J. Human resources for health and universal health coverage: progress, complexities and contestations. In: Rispel L, Padarath A, editors. *South African Health Review* 2018. Durban: Durban: Health Systems Trust; 2018; 2018. Available from: http://www.hst.org.za/publications/Pages/SAHR2018

8. Iwuji CC, Orne-Gliemann J, Larmarange J, Balestre E, Thiebaut R, Tanser F, et al. Universal test and treat and the HIV epidemic in rural South Africa: a phase 4, open-label, community cluster randomised trial. *Lancet HIV*. 2018 Mar;5(3):e116–25. Available from: https://linkinghub.elsevier.com/retrieve/pii/S2352301817302059

9. Fox MP, Rosen S, Geldsetzer P, Bärnighausen T, Negussie E, Beanland R. Interventions to improve the rate or timing of initiation of antiretroviral therapy for HIV in sub-Saharan Africa: meta-analyses of effectiveness. *J Int AIDS Soc*. 2016;19(1):20888. Available from: http://www.ncbi.nlm.nih.gov/pubmed/27507249

10. Zeglin RJ, Stein JP. Social determinants of health predict state incidence of HIV and AIDS: a short report. *AIDS Care.* 2015 Feb 16;27(2):255–9. Available from: http://www.ncbi.nlm.nih.gov/pubmed/25225050

11. Toska E, Pantelic M, Meinck F, Keck K, Haghighat R, Cluver L. Sex in the shadow of HIV: A systematic review of prevalence, risk factors, and interventions to reduce sexual risk-taking among HIV-positive adolescents and youth in sub-Saharan Africa. *PLoS One*. 2017;12(6):e0178106. Available from: http://www.ncbi.nlm.nih.gov/pubmed/28582428

12. Mabaso M, Sokhela Z, Mohlabane N, Chibi B, Zuma K, Simbayi L. Determinants of HIV infection among adolescent girls and young women aged 15–24 years in South Africa: a 2012 population-based national household survey. *BMC Public Health.* 2018 Dec 26;18(1):183. Available from: http://www.ncbi.nlm.nih.gov/pubmed/29373958

13. Statistics South Africa. Youth unemployment still high in Q1: 2018 | Statistics South Africa [Internet]. Available from: http://www.statssa.gov.za/?p=11129

14. Mutale W, Cleary S, Olivier J, Chilengi R, Gilson L. Implementing large-scale health system strengthening interventions: experience from the better health outcomes through mentoring and assessments (BHOMA) project in Zambia. *BMC Health Serv Res*. 2018 Dec 19;18(1):795. Available from: http://www.ncbi.nlm.nih.gov/pubmed/30340583

15. Mejia N, Perez-Arce F, Institute R, Lundberg M, Bank W, Munshi F, et al. Toward Solutions for Youth Employment [Internet]. Available from: http://www.ilo.org/wcmsp5/groups/public/---ed_emp/documents/publication/wcms_413826.pdf

16. The youth employment crisis: Time for action Fifth item on the agenda International Labour Office Geneva. International Labour Organization [Internet] [cited 2019 Jun 2]. Available from: www.ilo.org/publns.

17. Angwenyi V, Aantjes C, Kondowe K, Mutchiyeni JZ, Kajumi M, Criel B, et al. Moving to a strong(er) community health system: analysing the role of community health volunteers in the new national community health strategy in Malawi. *BMJ Glob Heal*. 2018 Nov 16;3(Suppl 3):e000996. Available from: http://www.ncbi.nlm.nih.gov/pubmed/30498595

18. Hill Z, Dumbaugh M, Benton L, Källander K, Strachan D, ten Asbroek A, et al. Supervising community health workers in low-income countries--a review of impact and implementation issues. *Glob Health Action*. 2014;7:24085. Available from: http://www.ncbi.nlm.nih.gov/pubmed/24815075

19. De Neve J-W, Boudreaux C, Gill R, Geldsetzer P, Vaikath M, Bärnighausen T, et al. Harmonizing community-based health worker programs for HIV: a narrative review and analytic framework. *Hum Resour Health*. 2017 Dec 3;15(1):45. Available from: http://www.ncbi.nlm.nih.gov/pubmed/28673361

20. Karimi-Shahanjarini A, Shakibazadeh E, Rashidian A, Hajimiri K, Glenton C, Noyes J, et al. Barriers and facilitators to the implementation of doctor-nurse substitution strategies in primary care: a qualitative evidence synthesis. *Cochrane Database Syst Rev*. 2019 Apr; 4:CD010412. Available from: http://www.ncbi.nlm.nih.gov/pubmed/30982950

21. Glenton C, Colvin CJ, Carlsen B, Swartz A, Lewin S, Noyes J, et al. Barriers and facilitators to the implementation of lay health worker programmes to improve access to maternal and child health: qualitative evidence synthesis. *Cochrane Database of Systematic Reviews*. 2013 Oct 8; 10: CD010414. Available from: http://www.ncbi.nlm.nih.gov/pubmed/24101553

22. Brown G, Reeders D, Cogle A, Madden A, Kim J, O'Donnell D. A Systems Thinking Approach to Understanding and Demonstrating the Role of Peer-Led Programs and Leadership in the Response to HIV and Hepatitis C: Findings From the W3 Project. *Front Public Heal*. 2018 Aug;6:231. Available from: http://www.ncbi.nlm.nih.gov/pubmed/30234083

23. Lewin S, Munabi-Babigumira S, Glenton C, Daniels K, Bosch-Capblanch X, van Wyk BE, et al. Lay health workers in primary and community health care for maternal and child health and the management of infectious diseases. *Cochrane Database Syst Rev*. 2010 Mar 17; Available from: http://doi.wiley.com/10.1002/14651858.CD004015.pub3

24. Kerry SM, Bland JM. Unequal cluster sizes for trials in English and Welsh general practice: implications for sample size calculations. *Stat Med*. 2001;20:377–90.
