# Supplementary material for: Impact of youth lay health workers on HIV service delivery in South Africa: A pragmatic cluster randomized trial of the Youth Health Africa program
Source: PLoS One. 2023 Nov 30;18(11):e0294719. doi: 10.1371/journal.pone.0294719 (PMC10688901; doi:10.1371/journal.pone.0294719)
Supplement: Attachment — Submitted filename: ResponseToReviewers_Tollefson_YHA_cRCT_Sept18.docx [file pone.0294719.s011.docx]

**RESPONSE TO REVIEWERS**

***MANUSCRIPT****:* ***Impact of youth lay health workers on HIV service delivery in South Africa: a pragmatic cluster randomized trial of the Youth Health Africa program***

**Reviewer #1:** This is an interesting pragmatic, cluster randomized trial among 20 facilities in Ngaka Modiri Molema district in Northwest province of South Africa over the period October 2020-August 2021 to assess impact of YHA interns on HIV testing, treatment initiation, and retention in care. The paper is generally well written and the Discussion articulated. However, there are some points than need to be further addressed.

**Major points**

**1. Was HIV-RNA available to measure? Unclear why this outcome was not considered for the treated in Table 1 besides retention in care.**

*Response:* Thank you for this question. This was a pragmatic trial that was entirely dependent on routinely collected data at the facility level for the facilities included in our analysis. Unfortunately, we did not have access to robust measures for HIV-RNA. However, we did include indicators from all three parts of the HIV cascade, as outlined in Table 1: testing (HTS_TST, HTS_POS), treatment (INITIATED_14DAYS), and retention in care (ART_DEFAULT_EARLY, ART_DEFAULT_LATE, ART_DEFAULT_ULTF). While retention in care does not measure viral suppression, it serves as a good proxy for engagement with the health system.

**2. As briefly discussed by the authors, power of the analysis is a concern. Researchers seems to have overestimated the underlying rate of HIV testing in these facilities. Indeed, they were expecting a rate of 18% while only 7% was the observed. This impacted on statistical power, regardless of the number of facilities involved.**

*Response:* Thank you for this comment. We agree that it was unfortunate that the analysis could not have greater power to detect change. Our initial power calculations suggested we would have sufficient power to detect change, but it would have been better to have more facilities engaged in the study (a point we address in the limitations section). Unfortunately, we were unable to recruit additional facilities to be in this study due to limitations of conducting a programmatic trial during the height of the COVID-19 pandemic.

While we agree that this study was underpowered, we would like to kindly address a misinterpretation of the numbers provided in your comment regarding the underlying rate of HIV testing at the facilities. At baseline, the control facilities saw 15.9% of people tested for HIV, while the intervention facilities saw 20.9% of people tested for HIV. The total proportion of people tested for HIV across both clinics was 18.4%. This is shown in Tables 2 and 3 in the manuscript. Power calculations were conducted based on the actual level of HIV testing at baseline, so we did not overestimate the underlying rate of HIV testing (at baseline) at these facilities.

You are correct in that we expected to see a greater change of HIV testing at facilities. In the section on Study Power (2.9), we state that “*we had 80% power to detect a minimum of 35% change for HIV testing (the primary outcome) if the intraclass correlation was 0.01 (i.e., change from 18.4% people tested to 24.5% of people tested), or a minimum of 82% change if the intraclass correlation was 0.05 (i.e., change from 18.4% of people tested to 33.4% of people tested); these calculations assumed a 5% level of significance.”* We saw a change from 15.9% to 22.4% in control clinics and a change from 20.9% to 32.6% in intervention clinics. While this was a large change, for both control and intervention groups, it was still a smaller change than our study was likely powered to observe.  **3. There is nothing particularly wrong with the analysis although the methodology used (difference in difference) is typical of observational studies in which there is no exchangeability at baseline. Although, some differences between intervention and controls facilities have been observed, none appeared particularly large, so it is unclear why a simpler analysis comparing the outcomes at the end of the study period comparing intervention and controls was not conducted.**

*Response:* Thank you for your comment. We agree that the difference-in-difference methodology is not commonly used for randomized trials—although it is used frequently when there end up being differences between groups at baseline, as was the case for our study. Prior to randomization, we had envisioned using a simple analysis comparing outcomes at the end of the study period. However, after randomization, we saw that the intervention and control clinics were not balanced with regards to the outcome measures we were going to be assessing (Table 2), with intervention clinics tending to have stronger measures at baseline (e.g., higher proportion of people tested for HIV). As such, a simple end-point analysis would risk overestimating the effect of the intervention. Conducting a difference-in-difference analysis was deemed a stronger analytic approach because it enabled us to be more conservative to further avoid erroneously rejecting the null hypothesis.

We conducted this pragmatic trial at the height of the COVID-19 pandemic in South Africa, and as such were not able to expand our sample size. If we had a larger number of clinics participating in the trial, the differences between clinics in the intervention and control groups would likely have been minor (or non-existent) at baseline and we could have relied upon a more traditional, and simpler, end-point analysis. In lieu of our inopportune circumstances, we believe the difference-in-difference methodology provides the most robust assessment of results for this study.

**4. I would avoid reporting p-values in Table 2. In addition, some of the reported figures are proportions. T-test is not a valid test to compare proportions.**

*Response:* Thank you for your comment. Per your suggestion, we have removed p-values from Table 2.  **5. I would have liked to see if control and intervention facilities were balanced with regards of admin interns at baseline (in terms of number interns/total head count at the facility) and a more formal comparison of attrition of interns over time (if data available).**

*Response:* Thank you for this comment. All facilities received 1-2 admin interns, depending on their needs (e.g., they could request the number of admin interns that they wanted to work at their facility). On average, control facilities had slightly more admin interns placed than intervention facilities (1.7 vs 1.4 admin interns per facility, respectively), but both control and intervention facilities had approximately 1 admin intern/clinic during the majority of the study period. In other words, there was slightly greater attrition in the control facilities than intervention facilities. We have added more information about the number of interns placed at the clinics and attrition within the text and in Supplement 2.

Section 3.2 in the manuscript now provides additional details on intern placement and attrition:

- *“Fifty interns were placed across twenty facilities in October 2020 (17 admin interns in control facilities; 14 admin interns and 19 program interns in intervention facilities).Thirty-four interns remained at 18 facilities as of August 2021 (12 in control facilities, 22 in intervention facilities).”*
- *“While intervention facilities received slightly more admin interns than intervention facilities (an average of 1.7 vs 1.4 interns/facility, respectively), attrition was greater in control clinics, leading to an equal number of admin interns serving at both intervention and control clinics over the study period. Please see Supplement 2 for more information on intern placement, attrition, and retention.”*

In particular, we would like to draw your attention to Supplemental Figures S2.1 and S2.2 and Tables S2.1 and S2.2; we have also pasted screenshots of below for your convenience.

For your additional information, we have provided the number of admin interns placed at each facility alongside the average monthly headcount at each facility (from the baseline period: January-August 2020) for you below. Facilities were free to request the number of interns they wanted, based on their needs and the specifications of the program/trial (e.g., control clinics could request 1 or 2 admin interns; intervention clinics could request 1 or 2 admin interns and 1 or 2 program interns). We have clarified this in the text in section 2.3 in the manuscript.

*“The exact number and roles of interns were determined by each facility, based on their needs and size.”*

| **Facility Number** | **ITT Group** | **As Treated Analysis** | **Average Monthly Headcount (Baseline: Jan - Aug 2020)** | **No. Admin Interns Placed** |
| --- | --- | --- | --- | --- |
| 1 | Control | Control | 2,340 | 2 |
| 2 | Control | Control | 1,455 | 2 |
| 3 | Control | Control | 569 | 2 |
| 4 | Control | Control | 1,573 | 1 |
| 5 | Control | Control | 875 | 2 |
| 6 | Control | Exclude | 1,373 | 2 |
| 7 | Control | Exclude | 1,414 | 2 |
| 8 | Control | Exclude | 1,131 | 1 |
| 9 | Control | Exclude | 1,573 | 1 |
| 10 | Control | Low | 1,085 | 2 |
| 11 | Intervention | High | 1,372 | 1 |
| 12 | Intervention | High | 715 | 2 |
| 13 | Intervention | High | 2,301 | 1 |
| 14 | Intervention | Low | 2,324 | 1 |
| 15 | Intervention | Low | 3,002 | 2 |
| 16 | Intervention | High | 719 | 1 |
| 17 | Intervention | High | 1,745 | 2 |
| 18 | Intervention | High | 1,737 | 1 |
| 19 | Intervention | Low | 1,792 | 2 |
| 20 | Intervention | Low | 1,613 | 1 |

**6. The key part of Tables 3,4 (DID analysis) is cut out from the pdf document. This is key for a correct interpretation as it shows the uncertainty around the DID estimates (not reported in the text).**

*Response:* Thank you for this note. Yes, we recognize these tables are cut off from a PDF version of the document. We are under the impression that we are following journal recommendations on how to include tables within the text; directions specify that we should include tables within the text, even if they are cut-off. It is our understanding that the editors will format the tables to fit on the page, post-acceptance. The tables can be fully seen in the 'Draft' layout when viewing the manuscript on Microsoft Word.

[Note: Here are the posted PLoS One guidelines for submitting tables with manuscripts: “*Do not split your table or otherwise try to make the table appear within the manuscript margins if it does not fit on one page. In Word, tables that run off of the manuscript page can be seen using Draft View…..*” ]

For your convenience, we have pasted the tables below as images, so that you can fully view them before the paper is formatted by journal editors.

 **7. Unclear whether the decision to look at the subsets of males adolescents was taken a priori or not. In any case it would have been advisable to formally test for interaction before showing the stratified results.**

*Response:* Thank you for this inquiry. The decision to look at the subset of males, adolescents, and male adolescents was made *a priori*. While this was considered a tertiary outcome for the study, program implementers were curious if YHA made a difference in HIV testing for these groups, as they are notoriously the most difficult to reach. We were only interested in HIV testing and not other outcomes, due to the challenges of reaching these populations with HIV tests. We have explained this in section 2.5 (Outcomes): *“In addition, we explored whether the YHA program increased testing among young people and males, two groups that are traditionally under-tested. To do this, we examined the proportion of people tested for HIV who were adolescents or young adults (ages 10-29 years old), the proportion of people tested for HIV who were male, and the proportion of people tested for HIV who were male adolescents or young adults.”*

We appreciate your comment to test for interaction, but we respectfully disagree about the need to do so. The analysis at hand was a simple comparison between the difference in proportions for HIV testing amongst the populations of interest between intervention and control clinics (Table 4) and was requested by program implementers. As observed in the results reported in Table 4, there were no differences in change in HIV testing between these two groups (net differences of approximately 0% for each category of interest). Given the utter lack of difference, the simple analysis at hand, and the tertiary nature of these outcomes, we do not believe there was a need to examine interactions before showing the stratified results.

**8. First paragraph of the Discussion/ final Conclusions. Because of the limited power I would be cautious in concluding that the intervention ‘had little impact’ or that it was a ‘null’ trial. Differences in some outcomes were quite large so it is possible that the trial was inconclusive due to limited power. No evidence for an effect does not mean no effect. The interpretation of the subset analysis is even more tricky as the study was not adequately powered to detect these in first place.**

*Response:* Thank you very much for this feedback. We agree that this study lacked sufficient power to provide robust inference on all the indicators we examined. We have updated the text in portions of the discussion and the conclusion to highlight this, while still being conservative in our interpretation of the results (e.g., referring to this as a largely ‘null’ trial).

Please note that we have highlighted in the Limitations and Conclusion that the study was underpowered to draw full inference on our results.

- *From the ‘Limitations’ section: “The limited number of facilities in this study coupled with the smaller level of change observed for HIV testing than initially expected resulted in this study being underpowered for the change observed in HIV testing; moreover, this study was not powered for the other outcomes we assessed, which limits the inference we can make on impact due to this intervention.”*
- *Portion of the ‘Conclusion’: “There were significant, immediate increases in HIV testing observed after program interns started in facilities, but there was insufficient evidence to draw robust inference on the program’s impact on improving indicators related to HIV positive cases, treatment initiation, retention in care, or testing among males or youth. While this was largely a null trial, we do not believe this suggests the YHA program had no impact on health facilities. Rather, when we review these results alongside previous research (12, 13), we believe study limitations, specifically intervention adherence and limited study power, could have muted or negated impact we observed and that this program should still be considered for roll-out.”*

However, we would also like to note that there were indicators (such as those in the tertiary age/sex analysis) that had point estimates for differences between the intervention and control group of ‘0’, which suggests that, even with limited power, there was likely no difference in outcomes based on how the intervention was rolled out in this study.

**Minor points**

**1. I would avoid words like ‘significant’, ‘insignificant’ etc. and rephrase in the context of evidence against the null hypothesis.**

*Response:* Thank you for this suggestion. As mentioned in our response above, we have attempted to rephrase aspects of the discussion and conclusion to highlight the differences that were found in this study—and that the study lacked sufficient evidence to reject the null hypothesis. For example, please see the Conclusion; portions relevant to this comment have been provided below.

*“There were significant, immediate increases in HIV testing observed after program interns started in facilities, but there was insufficient evidence to draw robust inference on the program’s impact on improving indicators related to HIV positive cases, treatment initiation, retention in care, or testing among males or youth. While this was largely a null trial, we do not believe this suggests the YHA program had no impact on health facilities. Rather, when we review these results alongside previous research (12, 13), we believe study limitations, specifically intervention adherence and limited study power, could have muted or negated impact we observed and that this program should still be considered for roll-out.”*

**2. Although the impact of quarantine/lockdown due to COVID-19 is a fair point, the sensitivity analysis restricted to facilities in which the interns were present throughout most of the study period should have mitigated this bias. The interference of COVID-19 care seems to be a more valid point.**

*Response:* Thank you for this comment. We agree that the general interference COVID-19 played on provision of healthcare likely impacted our results. We have added a sentence that addresses this in our discussion section:

*“Finally, the ebb and flow of COVID-19 may have affected patient access to care, which could have resulted in challenges for testing, treatment initiation, and retention in care amongst patients in both intervention and control clinics.”*

However, we also would like to note that we do think the amount of time that interns were able to be present at the facilities may have impacted results. While the sensitivity analysis differentiated clinics into “high intervention” or “low intervention” based on the overarching presence of interns at the facility, it did not capture routine absences. Program staff informed us that routine absences were higher during the COVID-19 period, due to the quarantine policy; unfortunately we do not have access to the exact number of days that each intern worked at the facility per month, only whether or not they were still employed and not on an extended leave of absence (e.g., greater than a month).

**3. Besides the fact that admin interns might have impacted on the rate of retention in care in both arms hence diluting the difference which could be other explanation for the larger difference between arms seen in rate of HIV testing alone? And why the waning over time?**

*Response:* As you noted, we saw a larger difference in HIV testing between the intervention and control arms of this study. We hypothesize this was because many programmatic interns were placed specifically to conduct HIV tests at facilities; these interns were often put in place to expand traditional testing efforts (not merely assume existing testing roles). As a result, in intervention facilities there was an immediate push to increase HIV testing—something control facilities did not have. This immediate increase can be seen well in the interrupted time series analysis (Figure 1A).

However, as you note, the difference in HIV testing between the intervention and control facilities waned over time; the level achieved at intervention facilities after placement of YHA interns remained constant after an initial jump, whereas testing gradually increased at the control facilities after the start of the program. We hypothesize this could be because intervention facilities reached peak testing performance after initial placement of the program interns and stayed at that level, whereas control facilities may have gradually decided to institute changes in their facilities to increase testing (as increasing testing is always a primary goal of PEPFAR).

**4. Some of the arguments in the main Discussion are repeated in the Limitation section (power, impact of COVID-19 etc). I would avoid these repetitions to improve reading flow.**

*Response:* Thank you for this note. We have rephrased and reduced portions of the limitations section to limit repetition.

**Reviewer #2:** The study quantified the impact of youth lay health workers on HIV service delivery in South Africa using a pragmatic cluster randomized trial.

These are my comments.

**1) Is the intervention Youth Health Africa (YHA) program or youth lay health workers on HIV. Is a bit confusing because in the abstract you indicated that you are assessing the impact of YHA interns on HIV testing, treatment initiation, and retention in care. The name of the intervention must be reflected in the title.**

***Response:*** Thank you so much for this comment. The intervention being assessed is called Youth Health Africa (YHA), and it is a youth lay health worker program. To help eliminate confusion, we have taken your advice and added the name of the intervention to the title. The title now reads: *Impact of youth lay health workers on HIV service delivery in South Africa: a pragmatic cluster randomized trial of Youth Health Africa.*

We would like to kindly point you to the portions of the Introduction where we describe the YHA program; we hope this helps further reduce confusion on the nature of the program.

*“Youth Health Africa (YHA) is an innovative lay health worker (LHW) approach that has showed potential to support HIV service delivery at health facilities in South Africa (12,13). YHA places young adults as temporary LHWs at health facilities in communities implementing the program to support non-clinical tasks, ranging from HIV testing and counseling to filing patient records (12). This program stands in contrast to traditional LHW programs in South Africa, as it operates as an internship program, with a primary goal to provide young adults workplace training to improve their future employability and reduce youth unemployment (11,14).”*

We also refer readers to two additional papers where we have described this intervention in detail:

- Tollefson D, Dasgupta S, Setswe G, Reeves S, Churchyard G, Charalambous S, and A Duerr. “Does a Youth Intern Program Strengthen HIV Service Delivery in South Africa? An Interrupted Time-Series Analysis.” *J Int AIDS Soc*. 2023; 26(4):e26083.
- Tollefson D, Ntombela N, Reeves S, Charalambous S, O’Malley G, Setswe G, and A Duerr.
  “ ‘‘They are gaining experience; we are gaining extra hands’: A mixed methods study to assess healthcare worker perceptions of a novel strategy to strengthen human resources for HIV in South Africa.” *BMC Health Serv Res.* 2023; 23(1): 27.

**2) Can you include the 95% confidence interval for the impact on HIV testing in the abstract. You only indicated 10.1% which is just the point estimate and doesn’t say much about the true effect size.**

*Response:* Thank you for this suggestion. We have edited the abstract to provide confidence intervals for HIV testing and treatment initiation. The results section of the abstract now reads as follows:

*“Fifty interns were placed in 20 facilities; thirty-four interns remained at 18 facilities through August 2021.Compared to control facilities, intervention facilities had a greater improvement in HIV testing (ΔΔ+5.2%, 95% Confidence Interval (CI): -4.6%-15.0%) and treatment initiation (ΔΔ+10.3%, 95% CI: -27.8-48.5%), but these differences were not statistically significant. There was an immediate increase in HIV testing in intervention facilities after program interns were placed, which was not observed in control facilities; this difference was significant (ΔΔ+10.1%, 95% CI: 1.5-18.7%, p=0.02). There were no other differences in outcomes observed between intervention and control facilities.”*

**3) Major comment: On what basis did you arrive at 20 health facilities (10 facilities in each arm)? Is your power analysis based on the number of health facilities (control versus intervention) or based on the number of individuals assigned to these health facilities. Since the intervention is creating clusters, your power analysis should be based on the number of health facilities not the number of individuals within the health facilities. We prefer more clusters (facilities randomized into intervention and controls) compared to the number of individuals within the facilities due to correlation. Within each facility, how many individuals (interns) were placed and what was the justification? This section is very scanty, and detail must be provided.**

*Response:* Thank you for this comment. We will address this comment in two parts: study power and the number of interns at facilities.

Study Power: We used the [“clusterPower” package in R](https://cran.r-project.org/web/packages/clusterPower/clusterPower.pdf) to complete our power calculations, which is designed to provide power calculations for all types of cluster randomized trials (cRCTs). As per standard power calculations for cRCTs, power calculations took into consideration both the number of health facilities and the number of people tested for HIV at these facilities. Full power calculations considered all of the following:

- The cluster size in each arm (i.e., the number of clinics)
- The mean cluster size (i.e., the number of people tested for HIV at clinic)
- Designated alpha level (i.e., 0.05)
- Designated power (i.e., 0.8 for 80% power)
- The coefficient of variation for number tested (i.e., cv)
- The intraclass correlation coefficient (i.e., icc)
- Baseline level of treatment (p1) (i.e., baseline level of HIV testing)
- Estimated level of treatment after intervention (p2) (i.e., endline level of HIV testing)

We agree that we would prefer to have more clusters, when possible. When envisioning this study, calculations suggested we could have between 18-24 facilities to achieve adequate power. Due to the pragmatic nature of this study and that it was implemented in the height of the COVID-19 pandemic, we identified 20 facilities that matched our inclusion criteria for participation in this study. Specifically, as COVID-19 delayed implementation of this study, many facilities had already received interns by the time the study was ready to be implemented; per our eligibility criteria for facilities, we could only include facilities that had not yet received any interns. This limited the total number of facilities that could participate in this trial; however, the total number of facilities was still within what we had originally envisioned and we believed a priori would provide sufficient power to examine the intervention.

Number of interns: Thank you for suggesting that we add more information about how interns were placed at facilities.

We describe how many interns were placed at facilities in two locations. Firstly, in section 2.3 (in the ‘Methods’ section), we describe the broad number of interns placed per facility and the rationale for these placements. After your comment, we have emphasized that “the exact number and roles of interns were determined by each facility, based on their needs and size.”

“*All 20 facilities received a minimum package of 1-2 interns assigned to administrative roles, such as filing or data capture (henceforth called “admin interns”). This helped to ensure consistent data quality between intervention and control facilities; this was necessary to ensure changes we observed were due to an impact on HIV service delivery and not an artefact of improvements in data quality. Intervention facilities each received the minimum package plus the intervention package: 1-2 interns assigned to support programmatic roles, like HIV testing and counseling, patient navigating, and tracing (henceforth called “program interns”). The exact number and roles of interns were determined by each facility, based on their needs and size.”*

Per your suggestion, we have now also provided additional details on the exact number of interns placed per facility in the ‘Results’. Section 3.2 in the manuscript now provides the following details on intern placement and attrition:

- *“Fifty interns were placed across twenty facilities in October 2020 (17 admin interns in control facilities; 14 admin interns and 19 program interns in intervention facilities).Thirty-four interns remained at 18 facilities as of August 2021 (12 in control facilities, 22 in intervention facilities).”*
- *“While intervention facilities received slightly more admin interns on than intervention facilities (an average of 1.7 vs 1.4 interns/facility, respectively), attrition was greater in control clinics, leading to an equal number of admin interns serving at both intervention and control clinics over the study period. Please see Supplement 2 for more information on intern placement, attrition, and retention.”*

Finally, we would like to draw your attention to Supplement 2, where we include trial monitoring data. Specifically, we have created a few new tables and figures—Supplemental Figures S2.1 and S2.2 and Tables S2.1 and S2.2—where we provide a summary of the average number of interns per facility and how this changed over the course of the study period. For your convenience, we have pasted screenshots of the new supplemental figures and tables below.

***4) You admitted that the 20 facilities were purposively selected which clearly means* that your study is not externally valid because the 20 facilities will not be representative of all target health facilities. Can the study be recommended for a larger scale up?**

*Response*: Thank you for your observation. We would like to highlight the selection criteria, which were used to purposefully select these facilities. Health facilities were eligible for this study if they were:

1. Located in Ngaka Modiri Molema district in North West province
2. Offered HIV services as part of routine clinic programs/operations
3. Had never received interns from YHA
4. Collaborated with Aurum Institute as a PEPFAR implementing partner
5. Had a need for three interns (the minimum number of interns that would be placed in a clinic assigned to the intervention group)
6. Interested in participating in the intervention

We agree that this makes the 20 facilities selected not representative of all target facilities, something that was unavoidable for the size and scope of this pragmatic trial. To ensure a comparable intervention and control group, it was necessary to choose facilities from a similar geographic area; moreover, this was a pragmatic trial, and we could only study facilities where the program was being implemented prospectively. We recognize that facilities in other provinces may have different needs than those in this one district in North West province. Furthermore, and perhaps most importantly, facilities with a demonstrated need for as many as three interns could be different than those who only had a demonstrated need for a single intern.

While we agree that this does limit the study’s external validity, we believe the evidence produced by this study in conjunction with the evidence produced by the other larger, quasi-experimental (interrupted time series) study highlighted in the discussion section suggests that this program could be recommended for scale-up—presuming it is offered at the right dose (e.g., sufficient interns are placed per facility).

We have clarified this in the conclusion:

*“While this was largely a null trial, we do not believe this suggests the YHA program had no impact on health facilities. Rather, when we review these results alongside previous research (12, 13), we believe study limitations, specifically intervention adherence and limited study power, could have muted or negated impact we observed and that this program should still be considered for roll-out. In particular, YHA may impact HIV service delivery if there are a high enough number of interns placed at the facility, which we may not have achieved in this study. Moving forward, there is a need to further elucidate the circumstances under which YHA provides its greatest impact and to reassess the indicators that should be used to measure the program’s impact on the health facility.”*

**5) You indicated that the study had 80% power to detect a minimum of 35% change for HIV testing if the intraclass correlation was 0.01 (i.e., change from 18.4% people tested to 24.5% of people tested), but a change of 18.4% people tested to 24.5% is like 6.1 percentage point increase, so why 35% change for HIV testing. Please explain.**

*Response***:** Thank you for this note. The 35% change referenced here is the proportional amount of change between the two measurements, i.e., the percentage increase when going from 18.4 to 24.5 (Calculation: (24.5-18.4)/18.4 = 33.2% change). The wording can be tricky, as we are examining changes in a percentage-based outcome—but stating the overarching percentage change. (Note: We recognize that we originally rounded to 35%; we adjusted the number in the text to reflect the more precise 33% change.)

**Results and discussions are okay on condition the point raised in the method section are addressed. They follow the CONSORT checklist.**

While revising your submission, please upload your figure files to the Preflight Analysis and Conversion Engine (PACE) digital diagnostic tool, [https://pacev2.apexcovantage.com/](https://urldefense.com/v3/__https:/pacev2.apexcovantage.com/__;!!GuAItXPztq0!jVD_OWpO5NgFfV-0a3MoG-Kth_khfwV2oPn6vStHZcy_cGjUlrdM3ualBKyrMtByVbV1KPxP1ikr5IZGDKk$). PACE helps ensure that figures meet PLOS requirements. To use PACE, you must first register as a user. Registration is free. Then, login and navigate to the UPLOAD tab, where you will find detailed instructions on how to use the tool. If you encounter any issues or have any questions when using PACE, please email PLOS at [figures@plos.org](mailto:figures@plos.org). Please note that Supporting Information files do not need this step.
